# Targeting Kinetoplastid Parasites with ProTide Prodrugs: A Proof‐of‐Concept Study

**DOI:** 10.1002/cmdc.202501072

**Published:** 2026-04-03

**Authors:** Silvester Lowe, Vishal Satikuvar, Tanjia M. Syeda, Monica Cal, Pascal Mäser, Marcel Kaiser, Hachemi Kadri

**Affiliations:** ^1^ Department of Pharmacy School of Life Sciences, Pharmacy and Chemistry Kingston University London UK; ^2^ Department of Medical Parasitology and Infection Biology Swiss Tropical and Public Health Institute (Swiss TPH) University of Basel Basel Switzerland

**Keywords:** cordycepin, kinetoplastids, neglected tropical diseases, nucleoside analogues, proTide

## Abstract

Neglected tropical diseases (NTDs) remain a major global health challenge, particularly in low‐ and middle‐income countries. Kinetoplastid parasites causing Chagas disease, leishmaniasis, and African trypanosomiasis rely on host purine salvage pathways, making nucleoside analogues attractive therapeutic candidates. However, their clinical utility is limited by poor cellular uptake and rapid metabolism. Herein, we report the application of the ProTide prodrug technology, a clinically validated approach that enhances the intracellular delivery of nucleoside monophosphates for the treatment of kinetoplastids infections. As a proof of concept, a focused library of zidovudine (AZT) and cordycepin ProTide prodrugs was designed, synthesized, and evaluated for antiparasitic activity against *T. b. rhodesiense*, *T. cruzi*, and *L. donovani*, as well as for cytotoxicity in L6 rat myoblasts. Out of these, compound **16** exhibited substantial serum stability and potent activity (IC_50_ = 5 nM; selectivity index, SI = 2,560) against *T. b. rhodesiense* with robust activity also observed against *T. cruzi* and *L. donovani.* These findings establish the ProTide prodrug technology as a promising strategy for optimizing nucleoside analogues against kinetoplastid parasites and provide a framework for the development of new therapeutics for NTDs.

## Graphical abstract

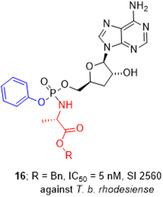

## 1 Introduction

Kinetoplastid protozoa are the causative agents of several neglected tropical diseases (NTDs), including Chagas disease, human African trypanosomiasis (HAT), and leishmaniasis [1, 2]. These infections are caused by *Trypanosoma cruzi*, *Trypanosoma brucei*, and *Leishmania* spp., respectively. A hallmark of kinetoplastids is the presence of a kinetoplast; a DNA‐rich structure within their single, large mitochondrion, which distinguishes them from other protozoan pathogens [3]. Endemic to tropical and subtropical regions, these parasites collectively affect millions of individuals annually [4]. Chagas disease, prevalent in Latin America, can progress to chronic cardiac and gastrointestinal complications if untreated [5]. HAT, or sleeping sickness, is fatal without intervention and presents in two forms depending on the *T. brucei* subspecies, with distinct clinical courses and treatment regimens [4]. Leishmaniasis manifests as cutaneous, mucocutaneous, or visceral disease, with visceral leishmaniasis being the most severe and potentially fatal form [6]. Despite progress in HAT control, including the introduction of safer oral agents such as fexinidazole and a sustained decline in reported cases, therapeutic gaps remain, and resistance is a concern [7, 8, 9, 10]. For Chagas disease and leishmaniasis, current therapies (e.g., benznidazole (BZN), nifurtimox, antimonials, amphotericin B, miltefosine (MTF), paromomycin) are limited by toxicity, prolonged regimens, variable efficacy in chronic or visceral disease, and emerging resistance [4, 11]. Nevertheless, persistent toxicity, limited efficacy in advanced or chronic disease, and a sparse clinical pipeline mean that discovery and development of new, safe, accessible drugs for kinetoplastid infections remain an urgent global health priority [12].

Kinetoplastid protozoa, unlike their mammalian hosts, lack the capacity for de novo purine biosynthesis and therefore dependent on host purine salvage via nucleoside/nucleobase transporters and dedicated enzymes [13, 14, 15]. In contrast, these parasites possess both de novo and salvage pathways for pyrimidines, enabling flexible utilization of exogenous or synthesized pyrimidine bases [16, 17]. Purine and pyrimidine‐based nucleoside analogues have thus emerged as promising candidates for the treatment of kinetoplastid infections, capitalizing on the parasites’ reliance on the host for nucleic acid biosynthesis [18, 19, 20, 21, 22, 23]. These compounds mimic natural nucleosides and disrupt DNA/RNA synthesis, ultimately resulting in parasite death. Numerous nucleoside analogues have been developed and evaluated in vitro against Chagas disease and leishmaniasis, as recently reviewed [24].

Clinical application of nucleoside analogues is frequently limited by poor cellular uptake, inefficient intracellular phosphorylation, and rapid dephosphorylation [25, 26, 27]. To address these drawbacks, various prodrug strategies of nucleoside monophosphates have been developed, most notably the aryloxy triester phosphoramidate prodrug technology, commonly known as the ProTide prodrug technology [28, 29, 30]. In this strategy, the phosphate group is masked with an aryl group and an amino acid ester, enhancing membrane permeability and bypassing the rate‐limiting first phosphorylation step. Upon cellular entry, enzymatic cleavage of the masking groups releases the active monophosphate, which is subsequently phosphorylated to the triphosphate form. The ProTide approach has been successfully used in drug discovery and has led to three FDA‐approved antiviral drugs, sofosbuvir, tenofovir alafenamide, and remdesivir [29, 30].

**FIGURE 1:**
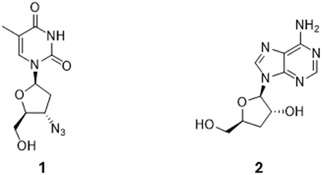
Chemical structures of AZT (**1**) and cordycepin (**2**).

**FIGURE 2:**
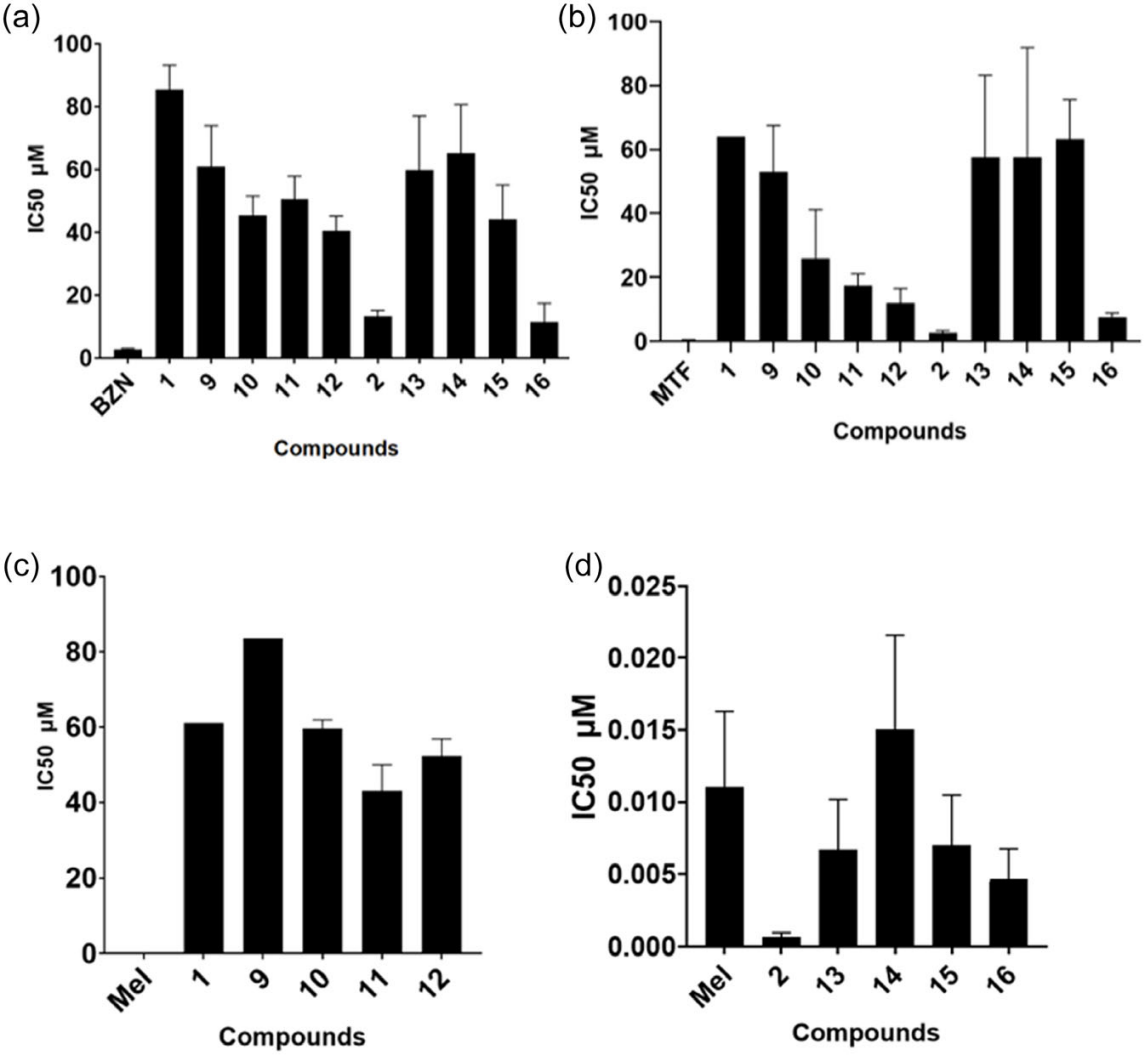
Antitrypanosomal activity of AZT (**1**), cordycepin (**2**), and their ProTide derivatives against (a) *T. cruzi*, (b) *L. donovani*, and (c,d) *T. b. rhodesiense*. IC50 values were determined from sigmoidal dose–response curves fitted with a four‐parameter logistic model in GraphPad Prism. Data points represent the mean ±SD of three independent experiments (*n* = 3).

Zidovudine (AZT; 3′‐azido‐3′‐deoxythymidine; Figure 1, **1**) is a synthetic pyrimidine nucleoside analogue originally developed as an antiretroviral agent, whereas cordycepin (3′‐deoxyadenosine; Figure 1, **2**) is a naturally occurring purine nucleoside analogue from Cordyceps species [31, 32]. Both compounds have demonstrated in vitro antiparasitic activity against kinetoplastids with potency varying between the species [24, 33, 34].

As these two nucleoside analogues are likely to suffer from the drawbacks highlighted above for nucleoside therapeutics, we applied the ProTide prodrug technology to AZT and cordycepin to enhance their antikinetoplastid activity. We designed and synthesized a focused library of ProTides and profiled their activity against *Trypanosoma brucei rhodesiense*, *Trypanosoma cruzi*, and *Leishmania donovani* alongside L6 cytotoxicity and serum stability.

## 2 Results and Discussion

### 2.1 Design and Synthesis of AZT and Cordycepin ProTides

The design of ProTide derivatives was guided by the need to enhance the uptake of the parent nucleoside analogues, AZT (**1**) and cordycepin (**2**), to maximize their antitrypanosomal efficacy. In the ProTide approach, the nucleoside monophosphate is masked with an aryl group and an amino acid ester, neutralizing the phosphate charge and increasing membrane permeability. A phenyl group and an *L*‐alanine ester were chosen as the aryl and amino acid components, respectively. This combination is widely used in ProTide chemistry and features in clinically approved drugs such as sofosbuvir and tenofovir alafenamide offering a favourable balance of stability and enzymatic activation [29, 35]. A focused set of ester groups methyl (Me), isopropyl (*i*Pr), *tert*‐butyl (*t*Bu), and benzyl (Bn) was selected to probe the influence of steric and electronic effects on biological activity and establish comparative structure–activity relationship trends across both purine and pyrimidine scaffolds. Upon cellular uptake, ProTides undergo enzymatic activation with carboxylesterase‐mediated cleavage of the ester followed by spontaneous amino acid elimination and phosphoramidase hydrolysis, releasing the corresponding nucleoside monophosphate [29, 36]. This bypasses the rate‐limiting initial phosphorylation of the parent nucleoside and underpins the design rationale of the series.

**SCHEME 1:**
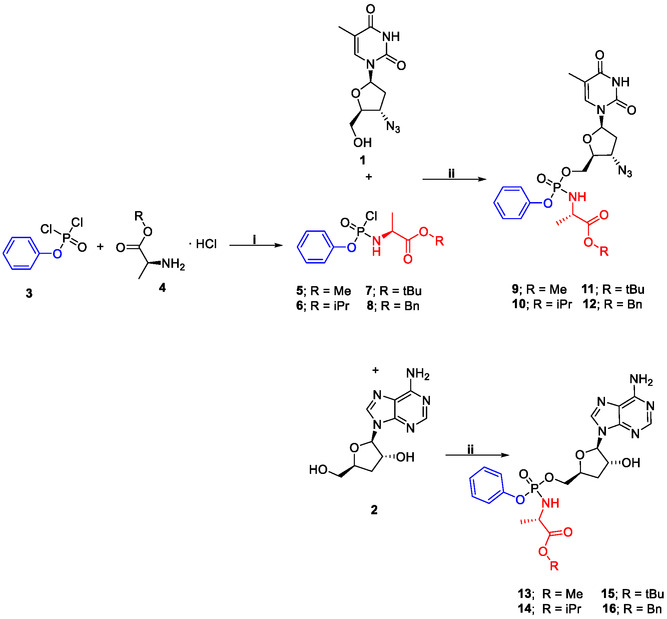
Synthesis of AZT and cordycepin ProTides. Reagents and conditions: (**i**) L‐alanine ester hydrochloride, TEA, DCM, −78°C for ½ hr then 2 h, rt, yields (76%–93%). (**ii**) NMI, THF, N_2_, 18 h, rt, yields (**9–12**, 46%–56%); (**13–16**, 22%–30%).

Synthesis followed established aryloxy triester phosphoramidate protocols employing N‐methylimidazole (NMI) as the coupling reagent, consistent with literature methods for ProTide prodrug preparation [37]. As shown in Scheme 1, the synthetic route involved two key steps. First, phenyl phosphorodichloridate was reacted with *L*‐alanine esters in anhydrous dichloromethane (DCM) and triethylamine (TEA) to generate the corresponding phosphorochloridate intermediates, obtained in 76%–93% yield. In the second step, these intermediates were coupled to the 5′‐hydroxyl group of **1** or **2** in the presence of NMI, affording the desired aryloxy triester phosphoramidates as diastereomeric mixtures due to the chiral phosphorus centre. Yields for the second step were higher for AZT derivatives (46%–56%) than cordycepin analogues (22%–30%) reflecting the increased purification challenges encountered for the latter compounds. The final products were purified by flash chromatography, and compound structures and purities were confirmed by ^1^H NMR, ^31^P NMR, HPLC, and high‐resolution mass spectrometry.

### 2.2 Antitrypanosmal Activity and Cytotoxity of Compounds

The antitrypanosomal activity of AZT (**1**), cordycepin (**2**), and their ProTide derivatives (**9–16**) was evaluated in vitro against *T. b. rhodesiense*, *T. cruzi*, and *L. donovani* using standardized microtiter assays with resazurin or colorimetric viability readouts. All assays were performed using validated protocols at the Swiss TPH [38, 39]. Melarsoprol (Mel), BZN, and MTF were included as positive controls with IC_50_ values of 0.011, 2.68, and 0.35 μM for the respective assays.

As shown in Figure 2, the parent nucleosides exhibited distinct activity profiles. AZT **(1)** exhibited poor activity across the three parasites (IC_50_ = 61 μM for *T. b. rhodesiense*, 85 μM for *T. cruzi*, and >64 μM for *L. donovani*). In contrast, cordycepin **(2)** exhibited more potent activity against *T. b. rhodesiense* (IC_50_ = 0.6 nM) than Mel (IC_50_ = 11 nM) and showed good low micromloar activity against *L. donovani* (IC_50_ = 2.66 μM) and moderate activity against *T. cruzi* (IC_50_ = 13 μM), demonstrating the superior intrinsic potency of the purine scaffold for activity against kinetoplastids.

Against *T. b. rhodesiense* (Figure 2c,d), cordycepin ProTide derivatives (**13–16**) maintained high potency, with all analogues active in the low nanomolar range. Compound **16** was the most potent (IC_50_ = 5 nM), approximately twofold more potent than Mel. In contrast, the designed ProTides (**10–12**) of AZT **(1)** resulted in modest improvements, with compound **11** showing the greatest activity (IC_50_ = 43 μM).

In *T. cruzi* (Figure 2a), cordycepin **(2)** exhibited better activity against AZT **(1)**, but ProTide derivatives of the latter **(9–12**) led to enhanced activity, with compound **12** exhibiting a twofold increase in potency (IC_50_ = 40 μM). On the other hand, ProTide derivatives of cordycepin (**13–16**) generally showed reduced activity, except for compound **16**, which retained similar potency to the parent nucleoside (IC_50_ = 11 μM). However, none of the compounds matched the potency of the positive control BZN (IC_50_ = 2.68 μM).

Against *L. donovani*, (Figure 2b), none of the compounds matched the potency of MTF (IC_50_ = 0.351 μM). Cordycepin **(2)** again demonstrated superior activity (IC_50_ = 2.66 μM) relative to AZT (**1**, IC_50_ = 64 μM). AZT‐based ProTides (**9–12**) improved potency, with compound **12** achieving an IC_50_ of 12 μM (5‐fold improvement). ProTides of cordycepin (**13–16**) generally resulted in reduced activity, except for compound **16** (IC_50_ = 7.6 μM).

Cytotoxicity was evaluated in the L6 rat myoblast cell line, with podophyllotoxin used as a positive control (IC_50_ = 0.031 μM) as shown in Table 1. The parent nucleosides exhibited distinct profiles: AZT **(1)** showed minimal cytotoxicity (IC_50_ > 100 μM), while cordycepin **(2)** displayed moderate cytotoxicity (IC_50_ = 36.3 μM). Cordycepin ProTides generally showed reduced cytotoxicity, with compounds **13**, **14**, and **15** exhibiting higher IC_50_ values (54.8, >100, and 57 μM, respectively) compared to the parent, except for compound **16**, which showed increased cytotoxicity (IC_50_ = 12.78 μM). Notably, despite its higher cytotoxicity, compound **16** maintained an excellent selectivity index for *T. b. rhodesiense* (SI = 2560), reflecting its potent antiparasitic activity. Similarly, AZT ProTide derivatives (**9–12**) maintained low cytotoxicity, with IC_50_ values remaining in the high micromolar range (63.8 – >100 μM).

**TABLE 1:** Cytotoxicity of AZT (1) and cordycepin (2) and their ProTides in L6 rat myoblast cell line.

Compound ID	**Cytotoxicity L6** **IC50, µM**	Compound ID	**Cytotoxicity L6** **IC50, µM**
**Podophyllotoxin**	0.031		
**1**	>100	**2**	36.33
**9**	97.2	**13**	54.76
**10**	>100	**14**	>100
**11**	86.9	**15**	57.6
**12**	63.8	**16**	12.78

**FIGURE 3:**
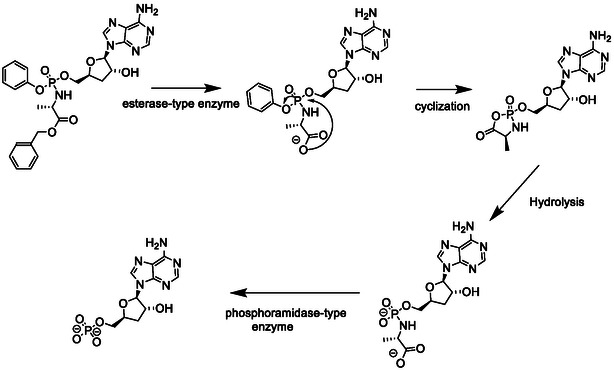
Proposed mechanism of intracellular activation of ProTide (**16**) in kinetoplastid parasites. The ProTide (masked monophosphate) enters the parasite via passive diffusion. Inside the cell, the benzyl ester moiety is cleaved by an esterase‐type enzyme, followed by spontaneous cyclization and displacement of the aryl group. The resulting intermediate is opened by water and then cleaved by a phosphoramidase‐type enzyme to release the active cordycepin monophosphate.

The ester group in the ProTide scaffold markedly influenced antiparasitic activity. Bn esters consistently yielded the highest potency across all parasite models, likely due to increased lipophilicity and efficient enzymatic activation [29]. Me and iPr esters provided intermediate activity, while tBu esters generally resulted in reduced potency, possibly as a result of increased steric hindrance. These trends were particularly evident in the cordycepin series, where the Bn ester analogue (**16**) was the most active, and in the AZT series, where Me and iPr esters moderately improved activity over the parent nucleoside. Cytotoxicity data further showed that AZT ProTide derivatives maintained low toxicity across all ester variants. In the cordycepin series, most ProTides (**13–15**) displayed reduced L6 toxicity relative to the parent, whereas the Bn ester (**16**) was more cytotoxic but nevertheless delivered an excellent selectivity index. This variation highlights the critical role of the initial hydrolysis step in ProTide activation. While mammalian activation is driven by carboxylesterase 1 (CES1) and cathepsin A (CatA) [29, 30], the specific esterase repertoire in kinetoplastids is less defined [4]. As proposed in Figure 3, the activation pathway initiates with an esterase‐mediated cleavage of the ester motif, which triggers a spontaneous intramolecular cyclization and subsequent phosphoramidase processing to release the active monophosphate. The superior activity of the benzyl ester suggests it may be a preferred substrate for parasite‐specific enzymes, whereas the bulkier tBu group may impede enzymatic cleavage. These findings suggest that careful ester selection can improve the activity–toxicity balance in ProTide design against kinetoplastids.

### 2.3 Stability Assay of Compound 16

**FIGURE 4:**
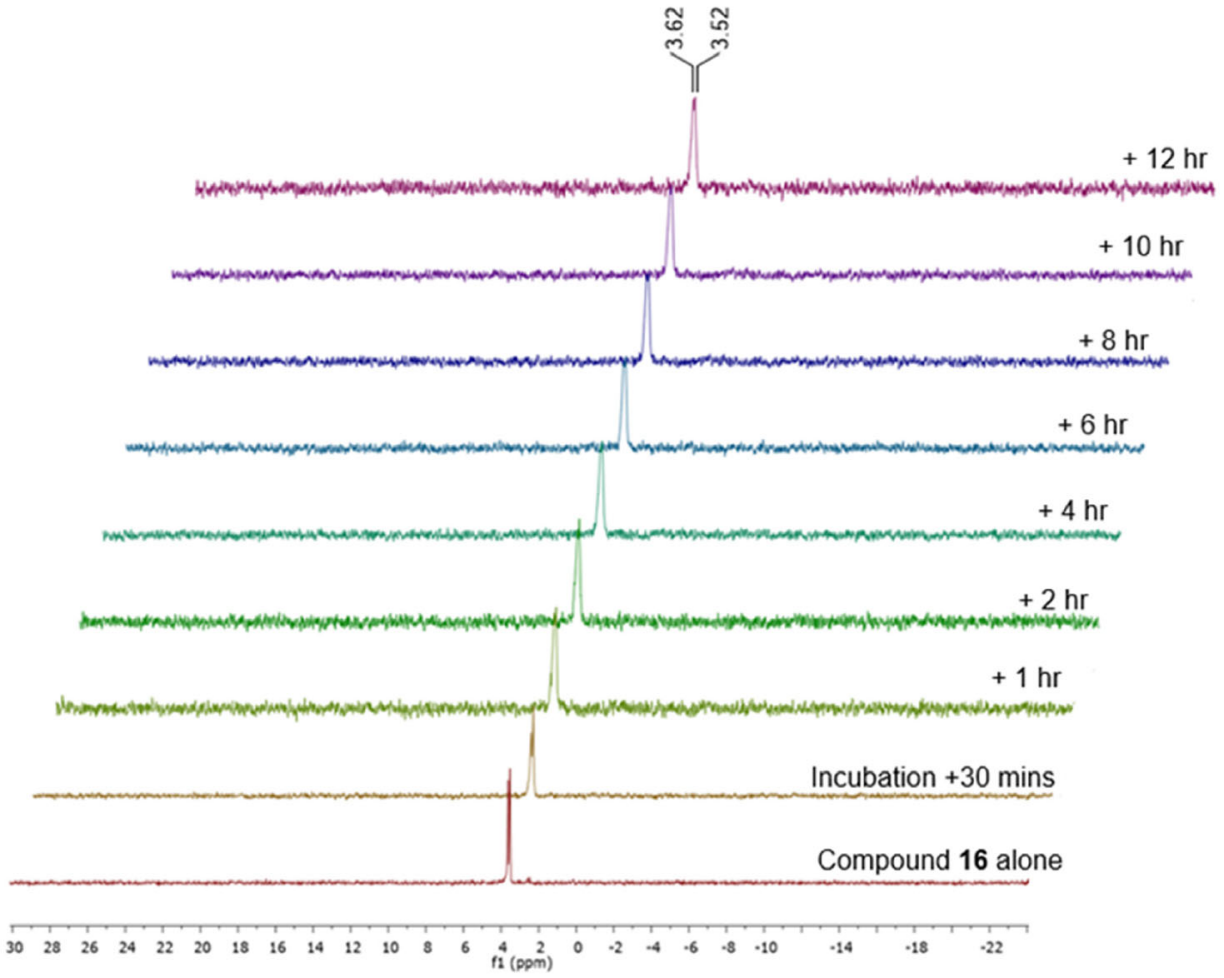
Stability of ProTide 16 in human serum at 37°C over 12 h as monitored by ^31^P NMR.

Cordycepin suffers from poor in vivo bioavailability due to rapid metabolism, leading to a shortened plasma half‐life and reduced systemic exposure [40]. Therefore, the metabolic stability of the most active compound, **16,** was evaluated by incubation in human serum at 37°C for 12 h, followed by analysis of the reaction mixtures by ^31^P NMR spectroscopy, as previously described [41, 42]. As shown in Figure 4, the ^31^P NMR spectrum of compound **16** exhibited the distinct chemical shifts characteristic of ProTide analogues. Notably, these signals remained unchanged throughout the 12‐h incubation period, and no additional signals were detected.

The substantial serum stability of analogue **16** supports its potential for further preclinical evaluation. Notably, this analogue corresponds to NUC‐7738 that has recently been investigated in oncology, where it demonstrated improved bioavailability and early clinical efficacy by protecting cordycepin from rapid deamination and bypassing transporter and kinase dependence [43]. Although distinct from our investigation, this clinical precedent demonstrates the translational viability of the ProTide approach and provides additional confidence in its potential clinical utility against kinetoplastid infections.

Building on these insights, future studies will need to focus on assessing the in vivo efficacy of compound **16** in relevant infection models and on expanding SAR exploration to optimize potency and selectivity. Crucially, these studies must also address the distinct therapeutic requirements for NTDs, where antiparasitic therapies for resource‐limited settings necessitate short‐course, highly tolerable regimens [4].

## 3 Conclusion

To our knowledge, this work presents the first use of the clinically validated ProTide prodrug technology to potentially improve the antiparasitic efficacy of nucleoside analogues against kinetoplastid NTDs. AZT‐based ProTides (**9–12**) exhibited improved, but rather limited, antiparasitic activity, most notably against *L. donovani*, while maintaining low cytotoxicity. In contrast, cordycepin ProTides (**13–16**) retained high potency against *T. b. rhodesiense*, with compound **16** combining potent antiparasitic activity (IC_50_ = 5 nM) and an excellent selectivity index (SI = 2560). The profile of **16** is particularly noteworthy given that it corresponds to NUC‐7738, which has been reported recently to demonstrate clinical safety and efficacy in a first‐in‐human Phase I trial for cancer. Collectively, these findings establish compound **16** as a promising lead for the development of effective therapeutics against *T. b. rhodesiense* and highlight the broader potential of ProTide technology to address unmet therapeutic needs in NTDs.

## 4 Experimental Section

### 4.1 General Information

Reagents used were purchased from Merck Life Sciences and Fluorochem and were used without further purification. All moisture‐sensitive reactions were carried out in oven‐dried round‐bottom flasks under an argon atmosphere in dry solvents. Analytical thin layer chromatography (TLC) was carried out on silica gel 60 F_254_ TLC plates. Flash chromatography was performed manually with 60 Å silica gel from Fluorochem or with an automated Teledyne CombiFlash NextGen100 using RediSep prepacked silica gel columns. High‐resolution mass spectra and liquid chromatography/mass spectrometry were acquired by ESI using a Q‐Tof Premier mass spectrometer (Waters). NMR spectra were recorded on Bruker Avance III‐HD‐400, Bruker Neo‐400, Varian DD2−500, Varian VNMRS‐600 and Varian VNMRS‐700 spectrometers. The chemical shifts (*δ*) are reported in parts per million (ppm), multiplicity as *s* = singlet, *d* = doublet, *t* = triplet, *q* = quartet, *m* = multiplet, coupling constant (J) in Hertz (Hz)**.**

### 4.2 Phenyl (methoxy‐L‐Alaninyl)phosphorochloridate (5)

Under argon, TEA (2.10 mL, 15.06 mmol) in anhydrous DCM (8 mL) was added dropwise to a vigorously stirred mixture of phenyl phosphodichloridate (1.12 mL, 7.50 mmol) and L‐alanine methyl ester hydrochloride (1.05 g, 7.50 mmol) in anhydrous DCM (20 mL) at −70°C and the mixture was stirred for 30 min. The reaction was then left to stir for 3hr at room temperature, using ^31^P NMR to monitor the reaction. After completion, the solvent was removed *in vacuo,* and the residue was treated with diethyl ether (15 mL) and filtered, and solvent was evaporated *in vacuo* to yield a brown oil which was purified using flash chromatography (ethyl acetate: hexane, 7:3) to yield the product as a colourless oil (1.93 g, 93%). ^31^P NMR (162 MHz, CDCl_3_) δ 7.99, 7.63; ^1^H NMR (400 MHz, CDCl_3_) δ 7.45–7.35 (2H, m, Ar‐*H*), 7.32–7.23 (3H, m, Ar‐*H*), 4.46–4.10 (1H, m, N‐*H*, 1H, m, C*H*CH_3_), 3.83, 3.81 (2H, 2 s, OC*H*_3_), 1.52 (3H, dd, *J* = 7.1, 4.1 Hz, CHC*H*_3_). ^13^C NMR (100 MHz, CDCl_3_) δ 20.49 (CH*C*H_3_), 50.40 (*C*HCH_3_), 52.80 (O*C*H_3_), 120.56 (Ar‐*C*H), 126.00 (Ar‐*C*H), 129.92 (Ar‐*C*H), 149.76 (*C*OCH_3_), 173.15 (*C*OCH_3_).

### 4.3 Phenyl (isopropoxy‐L‐Alaninyl)phosphorochloridate (6)

Prepared as described for **5** using L‐alanine isopropyl ester hydrochloride (750 mg, 4.47 mmol), phenyl phosphorodichloridate (0.67 mL, 4.47 mmol), and Et_3_N (1.25 mL, 8.94 mmol). Colourless oil (1.03 g, 77%). ^31^P NMR (162 MHz, CDCl_3_) δ 8.10, 7.69; ^1^H NMR (400 MHz, CDCl_3_) δ 7.49–7.35 (2H, m, Ar‐*H*), 7.33–7.24 (3H, m, Ar‐*H*), 5.15–5.01 (1H, m, OC*H* ^i^Pr), 4.37–4.27 (1H, m, N‐*H*), 4.20–4.04 (1H, m, C*H*CH_3_), 1.50–1.47 (3H, m, CHC*H*_3_), 1.32– 1.23 (6H, m, OCH^*i*^ *Pr*). ^13^C NMR (100 MHz, CDCl_3_) δ 20.42 (CH*C*H_3_), 21.64 (2C, OCH^*i*^ *Pr*), 50.71 (*C*HCH_3_), 69.73 (O*C*H^i^Pr), 120.53 (Ar‐*C*H), 125.91 (Ar‐*C*H), 129.89 (Ar‐*C*H), 149.82 (Ar‐*C*OP), 172.10 (*C*OCH_3_).

### 4.4 Phenyl (tert‐Butoxy‐L‐Alaninyl)phosphorochloridate (7)

Prepared as described for **5** using L‐alanine tert‐butyl ester hydrochloride (820 mg, 4.51 mmol), phenyl phosphorodichloridate (0.66 mL, 4.51 mmol), and Et_3_N (1.25 mL, 8.97 mmol). Colourless oil (1.18 g, 83%). ^31^P NMR (162 MHz, CDCl_3_) δ 8.24, 7.81; ^1^H NMR (400 MHz, CDCl_3_) δ 7.42–7.33 (2H, m, Ar‐*H*), 7.30–7.20 (3H, m, Ar‐*H*), 4.35–4.16 (1H, m, N‐*H*), 4.15–3.96 (1H, m, C*H*CH_3_), 1.51–1.36 (3H, m, CHC*H*_3_, 9H, m, OC^*t*^ *Bu*). ^13^C NMR (100 MHz, CDCl_3_) δ 20.53 (CH*C*H_3_), 27.92 (OC^*t*^ *Bu*), 51.26 (*C*HCH_3_), 82.63 (O*C* ^t^Bu), 120.56 (Ph‐*C*H), 125.92 (Ph‐*C*H), 129.89 (Ph‐*C*H), 149.08 (Ph‐*C*OP), 171.98 (*C*OCO^t^Bu).

### 4.5 Phenyl (benzyloxy‐L‐Alaninyl)phosphorochloridate (8)

Prepared as described for **5** using L‐alanine benzyl ester hydrochloride (950 mg, 4.40 mmol), phenyl phosphorodichloridate (0.66 mL, 4.40 mmol), and Et_3_N (1.23 mL, 8.80 mmol). Colourless oil (1.18 g, 76 %). ^31^P NMR (162 MHz, CDCl_3_) δ 7.90, 7.53; ^1^H NMR (400 MHz, CDCl_3_) δ 7.46–7.30 (m, 5H, Ph‐*H*), 7.29–7.20 (m, 5H, Ph‐*H*), 5.21 (2H, d, *J* = 6.2 Hz, OC*H*_2_Ph), 4.35–4.07 (2H, 2 m, C*H*CH_3_), 1.52 (3H, dd, *J* = 6.8, 2.4 Hz, CHC*H*_3_).^13^C NMR (100 MHz, CDCl3) δ 20.51(CH*C*H_3_), 50.53 (*C*HCH_3_), 67.58 (O*C*H_2_Ph), 120.54 (Ph‐*C*H), 126.99 (Ph‐CH), 128.30 (Ph‐*C*H), 128.61(Ph‐*C*H), 128.66 (Ph‐*C*H), 129.94 (Ph‐*C*H), 135.04 (Ph‐*C*H), 149.74 (Ph‐*C*H), 172.50 (*C*OOCH_2_Ph)

### 4.6 Methyl (2S)‐2‐[[[(2S, 3S,5R)‐3‐Azido‐5‐(5‐Methyl‐2,4‐Dioxo‐Pyrimidin‐1‐Yl)tetrahydrofuran‐2‐Yl]methoxy‐Phenoxy‐Phosphoryl]amino]propanoate (9)

AZT **(1)** (280 mg, 1.05 mmol) was suspended in anhydrous THF (15 mL) under argon. To this, NMI (0.17 mL, 2.10 mmol) was then added dropwise over 15 min. The resulting solution was stirred for 10 min, and following this, compound **5** (348 mg, 1.25 mmol) in anhydrous THF (10 mL) was added dropwise over 10 min. The mixture was left to stir for 18 h. Solvent was removed under reduced pressure to leave the crude product as a pale‐yellow oil. This was purified via flash column chromatography (eluent 2:98 to 10:90 MeOH/CH_2_Cl_2_) to yield the desired product as a white solid (274 mg, 52%), ^31^P NMR (162 MHz, MeOD) δ 4.05, 3.77; ^1^H NMR (700 MHz, MeOD) δ 7.48 (d, *J* = 11.14 Hz, 1H, H‐6), 7.27–7.17 (m, 5H, Ph), 6.19–6.12 (m, 1H, H‐1′), 4.47–4.25 (m, 3H, 2 x H‐5′ and H‐4′), 4.08–3.86 (m, 1H, H‐3′), 3.89–3.85 (m, 1H, CH‐N), 3.70–3.63 (m, 3H, OCH_3_), 2.48–2.39 (m, 2H, H‐2′) 1.73–1.71 (dd, 3H, *J* *=* 1.26, 7.31 Hz, CH_3_), 1.34–1.24 (m, 3H, CH_3_).^13^C NMR (176 MHz, MeOD) δ 11.30 (CH_3_), 19.00 (CH_3_), 36.00 (CH_2_‐2′), 50.90 (CH), 51.40 (CH‐3′), 61.20 (CH2‐5′), 66.70 (CH2), 82.60 (CH‐4′), 85.35 (CH‐1′), 110.80 (C), 120.36 (Ph‐CH), 124.52 (Ph‐CH), 129.89 (Ph‐CH), 136.25 (CH), 150.14 (Ph‐C), 162.00 (*C*ON), 174.05 (*C*OOCH3). HRMS‐ESI (m/z): calcd for C_20_H_26_N_6_O_8_P [M + H]^+^ 509.1550 found 509.1552. HPLC CH_3_CN/H_2_O 5:95 to 95:5 in 30 min, *λ* = 254 nm, *t*_R_ = 16.33, 16.46 min.

### 4.7 Isopropyl (2S)‐2‐[[[(2S, 3S,5R)‐3‐Azido‐5‐(5‐Methyl‐2,4‐Dioxo‐Pyrimidin‐1‐Yl)tetrahydrofuran‐2‐Yl]methoxy‐Phenoxy‐Phosphoryl]amino]propanoate (10)

Prepared as described for **9** using AZT (280 mg, 1.05 mmol) and phenyl isopropoxyalaninyl phosphochloridate **6** (382 mg, 1.25 mmol). White solid (312 mg, 56%), ^31^P NMR (162 MHz, MeOD) δ 4.10, 3.81; ^1^ H NMR (700 MHz, MeOD) δ 7.46 (d, *J* = 11.02 Hz, 1H, H‐6), 7.27–7.08 (m, 5H, Ph,), 6.16–6.11 (m, 1H, H‐1′), 4.95 (m, 1H, CHiPr), 4.43–4.24 (m, 3H, 2 x H‐5′, H‐4′), 4.12 (m, 1H, H‐3′), 3.87–3.81 (m, 1H, CH‐N), 2.46–2.05 (m, 2H, H‐2′), 1.95–1.89 (dd, 3H, *J* *=* 1.23, 7.24 Hz, CH_3_), 1.32–1.23 (m, 3H, CH_3_), 1.18 (m, 6H, iPr); ^13^C NMR (176 MHz, MeOD) δ 12.00 (CH_3_), 18.35 (CH_3_), 22.65 (2 × CH_3_, iPr), 37.40 (CH), 49.21 (CH‐2′), 54.30 (CH‐3′), 61.22 (CH_2_), 66.00 (CH_2_‐5′), 69.33 (CH), 82.00 (CH‐4′), 84.50 (CH‐1′), 110.70 (CH), 120.36 (Ph‐CH), 124.52 (Ph‐CH), 129.00 (Ph‐CH), 138.46 (CH), 151.18 (Ph‐C), 162.56 (*C*ON). HRMS‐ESI (m/z): calcd for C_22_H_30_N_6_O_8_P [M + H]^+^ 537.1863 found 537.1848. HPLC CH_3_CN/H_2_O 5:95 to 95:5 in 30 min, *λ* = 254 nm, *t*_R_ = 18.37, 18.55 min.

### 4.8 Tert‐Butyl (2S)‐2‐[[[(2S, 3S,5R)‐3‐Azido‐5‐(5‐Methyl‐2,4‐Dioxo‐Pyrimidin‐1‐Yl) Tetrahydrofuran‐2‐Yl]methoxy‐Phenoxy‐Phosphoryl]amino]propanoate (11)

Prepared as described for **9** using AZT (280 mg, 1.05 mmol) and phenyl tert‐butyloxy‐L‐alaninyl phosphorochloridate **7** (400 mg, 1.25 mmol). White solid (270 mg, 47%). ^31^P NMR (162 MHz, MeOD) δ 4.13, 3.87; ^1^H NMR (700 MHz, MeOD) δ 7.44 (d, *J* = 11.10, 1H, H‐6), 7.29–7.10 (m, 5H, Ph), 6.17–6.12 (m, 1H, H‐1′), 4.44–4.23 (m, 3H, 2 x H‐5′ and H‐4′), 4.18–4.13 (m, 1H, H‐3′), 3.82–3.80 (m, 1H, CH‐N), 2.48–2.24 (m, 2H, H‐2′), 1.83–1.78 (dd, 3H, *J* = 1.29, 7.24 Hz, CH_3_), 1.42 (s, 9H, tBu), 1.32–1.24 (m, 3H, CH_3_);^13^C NMR (176 MHz, MeOD) δ 12.25 (CH_3_), 19.12 (CH_3_), 26.90 (3×CH_3_‐tBu), 36.10 (CH_2_‐2′), 48.31 (CH), 53.21 (CH‐3′), 61.71 (CH_2_), 64.27 (CH_2_‐5′), 78.06 (CH), 81.25 (CH‐4′), 82.62 (CH‐1′), 110.57 (CH), 120.01 (Ph‐CH), 124.26 (Ph‐CH), 128.76 (Ph‐CH), 138.82 (CH), 151.37 (C), 164.03 (*C*ON), 173.42 (*C*OOCH_3_). HRMS‐ESI (m/z): calcd for C_23_H_32_N_6_O_8_P [M + H]^+^ 551.2019 found 551.2001. HPLC CH_3_CN/H_2_O 5:95 to 95:5 in 30 min, *λ* = 254 nm, *t*_R_ = 19.14 min.

### 4.9 Benzyl (2S)‐2‐[[[(2S, 3S,5R)‐3‐Azido‐5‐(5‐Methyl‐2,4‐Dioxo‐Pyrimidin‐1‐Yl)tetrahydrofuran‐2‐Yl]methoxy‐Phenoxy‐Phosphoryl]amino]propanoate (12)

Prepared as described for **9** using AZT (280 mg, 1.05 mmol) and phenyl(benzyloxy‐L‐alaninyl)phosphorochloridate **8** (443 mg, 1.25 mmol). White solid (285 mg, 46%). ^31^P NMR (162 MHz, MeOD) δ 4.07, 3.65; ^1^H NMR (700 MHz, MeOD) δ 7.42 (d, *J* = 11.00 Hz, 1H, H‐6), 7.35–7.13 (m, 10H, 2x Ph), 6.17–6.09 (m, 1H, H‐1′), 5.12 (m, 2H, PhCH_2_), 4.34–4.22 (m, 3H, 2 × H‐5′ and H‐4′), 4.18–3.87 (m, 2H, CH‐N, H‐3′), 2.36–2.27 (m, 2H, H‐2′),1.78 (dd, 3H, *J* = 1.27, 7.29 Hz, CH_3_), 1.35–1.26 (m, 3H, CH_3_).^13^C NMR (176 MHz, MeOD) δ 12.30 (CH_3_), 18.10 (CH_3_), 37.30 (CH_2_‐2′), 52.10 (CH), 54.40 (CH‐3′), 60.19 (CH_2_), 64.70 (CH_2_‐5′), 67.30 (CH_2_), 82.13 (CH‐4′), 86.50 (CH‐1′), 110.70 (CH), 119.38 (Ph‐CH), 123.92 (Ph‐CH), 127.20 (Ph‐CH), 128.10 (Ph‐CH), 128.74 (Ph‐CH), 131.14 (Ph‐CH), 137.25 (C), 151.11 (CH), 151.23 (C), 162.11 (*C*ON), 174.21 (*C*OOCH_3_). HRMS‐ESI (m/z): calcd for C_26_H_30_N_6_O_8_P [M + H]^+^ 585.1863 found 585.1868. HPLC CH_3_CN/H_2_O 5:95 to 95:5 in 30 min, *λ* = 254 nm, *t*_R_ = 19.75, 19.87 min.

### 4.10 Methyl (2S)‐2‐[[[(2S, 4R,5R)‐5‐(6‐Aminopurin‐9‐Yl)‐4‐Hydroxy‐Tetrahydrofuran‐2‐Yl]methoxy‐Phenoxy‐Phosphoryl]amino]propanoate (13)

Prepared as described for **9** using cordycepin **2** (280 mg, 1.05 mmol) and phenyl methoxyalaninyl phosphochloridate **5** (350 mg, 1.25 mmol). White solid (142 mg, 28%). ^31^P NMR (162 MHz, MeOD) δ 3.95, 3.47; ^1^ H NMR (700 MHz, MeOD) δ 8.28 (d, 1H, *J* = 6.26 Hz, CH), 8.22 (s, 1H, CH), 7.35–7.16 (m, 5H, Ph), 6.03 (d, 1H, *J* = 6.08 Hz, H‐1′), 4.75 (m, 1H, H‐2′), 4.71 (m, 1H, H‐4′), 4.47–4.31 (m, 2H, H‐5′), 3.95–3.92 (m, 1H, CH‐N), 3.64 (m, 3H, OCH_3_), 2.39–2.11 (m, 2H, H‐3′), 1.30 (m, 3H, CH_3_);^13^C NMR (176 MHz, MeOD) δ 174.09 (*C*OOCH_3_), 155.93 (CH), 152.45 (CH), 150.73 (C), 148.82 (C), 139.07 (CH), 129.33 (Ph‐CH), 124.72 (Ph‐CH), 119.99 (Ph‐CH), 119.07 (Ph‐C), 91.77 (CH‐1′), 79.98 (CH‐ 4′), 75.18 (CH‐ 2′), 66.80 (CH_2_‐5′), 51.28 (CH_3_, C‐10), 50.23 (CH, C‐7), 33.38 (CH_2_, C‐13), 18.91 (CH_3_, C‐8). HRMS‐ESI (m/z): calcd for C_20_H_26_N_6_O_7_P [M + H]^+^ 493.1601 found 493.1588, HPLC CH_3_CN/H_2_O 5:95 to 95:5 in 30 min, *λ* = 254 nm, *t*_R_ = 12.51, 13.00 min.

### 4.11 Isopropyl (2S)‐2‐[[[(2S, 4R,5R)‐5‐(6‐Aminopurin‐9‐Yl)‐4‐Hydroxy‐Tetrahydrofuran‐2‐Yl]methoxy‐Phenoxy‐Phosphoryl]amino]propanoate (14)

Prepared as described for **9** using cordycepin **2** (270 mg, 1.00 mmol) and phenyl isopropoxyalaninyl phosphochloridate **6** (380 mg, 1.25 mmol). White solid (160 mg, 30%). ^31^P NMR (162 MHz, MeOD) δ 3.95, 4.77; ^1^H NMR (700 MHz, MeOD) δ 8.22 (d, 1H, *J* = 5.48 Hz, CH), 8.18 (s, 1H, CH), 7.23–7.03 (m, 5H, Ph), 5.90 (d, 1H, *J* = 4.02 Hz, H‐1′), 4.82 (m, 1H, CHiPr), 4.71–4.62 (m, 2H, H‐2′, H‐4′), 4.27–4.21 (m, 2H, H‐5′), 3.80 (m, 1H, CH‐N), 2.26‐ 2.00 (m, 2H, H‐3′), 1.17 (m, 3H, CH_3_), 1.05 (m, 6H, iPr); ^13^C NMR (176 MHz, MeOD) δ 173.17 (*C*OOCH_3_), 155.92 (C), 152.46 (CH,), 150.74 (C), 148.80 (C), 139.12 (C), 129.33 (Ph‐CH), 124.73 (Ph‐CH), 120.01 (Ph‐CH), 119.09 (Ph‐C), 78.49 (CH‐ 4′), 74.70 (CH‐2′), 68.70 (CH), 67.69 (CH_2_), 50.31 (CH), 33.33 (CH_2_‐3′), 20.60 (CH_3_), 19.13 (CH_3_, iPr); HRMS‐ESI (m/z): calcd for C_22_H_30_N_6_O_7_P [M + H]^+^ 521.1914 found 521.1908; HPLC CH_3_CN/H_2_O 5:95 to 95:5 in 30 min, *λ* = 254 nm, *t*_R_ = 14.73, 15.00 min.

### 4.12 Tert‐Butyl (2S)‐2‐[[[(2S, 4R,5R)‐5‐(6‐Aminopurin‐9‐Yl)‐4‐Hydroxy‐Tetrahydrofuran‐2‐Yl]methoxy‐Phenoxy‐Phosphoryl]amino]propanoate (15)

Prepared as described for **9** using cordycepin **2** (270 mg, 1.00 mmol) and phenyl tert‐butoxyalaninyl phosphochloridate **7** (640 mg, 2.00 mmol). White solid, (120 mg, 22%). ^31^P NMR (162 MHz, MeOD) δ 4.07, 3.96*;* ^1^H NMR (700 MHz, MeOD δ 8.27 (d, 1H, *J* = 5.26 Hz, CH), 8.19 (s, 1H, CH), 7.23–7.02 (m, 5H, Ph), 5.91 (d, 1H, *J* = 4.55 Hz, H‐1′), 4.64 (m, 1H, H‐2′), 4.61 (m, 1H, H‐4′), 4.34–4.20 (m, 2H, H‐5′), 3.79 (m, 1H, CH‐N), 2.26–2.01 (m, 2H, H‐3′), 1.39 (s, 9H, t‐Bu), 1.25 (m, 3H, CH_3_); ^13^C NMR (176 MHz, MeOD) δ 155.92 (C), 152.45 (CH), 139.11 (CH), 129.33 (Ph‐CH), 129.30 (Ph‐CH), 124.75 (Ph‐CH), 120.01 (Ph‐CH), 91.98 (CH‐1′), 81.14 (C), 78.95 (CH‐ 4′), 75.49 (CH‐ 2′), 66.87 (CH_2_‐ 5′), 50.71 (CH), 33.29 (CH_2_‐ 3′), 26.75 (CH_3_, *t*Bu), 19.18 (CH_3_); HRMS‐ESI (m/z): calcd for C_23_H_32_N_6_O_7_P [M + H]^+^ 535.2070 found 535.2082; HPLC CH_3_CN/H_2_O 5:95 to 5:95 in 30 min, *λ* = 254 nm, *t*_R_ = 15.72, 15.89 min.

### 4.13 Benzyl (2S)‐2‐[[[(2S, 4R,5R)‐5‐(6‐Aminopurin‐9‐Yl)‐4‐Hydroxy‐Tetrahydrofuran‐2‐Yl]methoxy‐Phenoxy‐Phosphoryl]amino]propanoate (16)

Prepared as described for **9** using cordycepin **1** (270 mg, 1.00 mmol) and phenyl benzoxyalaninyl phosphochloridate **8** (443 mg, 1.25 mmol). White solid (140 mg, 25%) ^31^P NMR (162 MHz, MeOD) δ 4.05, 3.73; ^1^H NMR (600 MHz, MeOD) δ 8.25 (d, *J* = 6.16 Hz, 1H, CH), 8.21(s, 1H,CH), 7.33‐ 7.13 (m, 10H, 2 x Ph), 5.98 (d, *J* = 9.55 Hz, 1H, H‐1′), 5.09 (m, 2H, PhCH_2_), 4.69 (m, 1H, H‐2′), 4.64(m, 1H, H‐4′), 4.40–4.24 (m, 2H, H‐5′), 3.96 (m, 1H, CH‐N), 2.25–2.05 (m, 2H, H‐3′), 1.30 (m, 3H, CH_3_);^13^C NMR (176 MHz, MeOD) δ 173.43 (*C*OOCH_3_), 155.89 (C), 152.44 (CH), 150.66 (C), 148.75 (C), 139.00 (CH), 135.82 (Ph‐C), 129.36 (Ph‐CH), 128.18 (Ph‐CH), 127.88 (Ph‐CH), 124.75 (CH, Ph‐CH), 120.05 (CH, C‐ Ph‐CH), 119.96 (CH, Ph‐CH), 119.13 (Ph‐C), 91.78 (CH, 1′), 79.08 (CH, 4′), 75.27 (CH, 2′), 66.83 (CH2), 66.54, 67.16 (CH_2_), 33.32 (CH_2_,), 18.98 (CH_3_); HRMS‐ESI (m/z): calcd for C_26_H_30_N_6_O_7_P [M + H]^+^ 569.1914 found 569.1921, HPLC CH_3_CN/H_2_O 5:95 to 95:5 in 30 min, *λ* = 254 nm, *t*_R_ = 16.31, 16.45 min.

### 4.14 Stability Assay

The method was based on the approach from references 41 and 42. ProTide **16** weighing 5.0 mg, was mixed with DMSO‐d6 (0.10 mL) and D_2_O (0.15 mL). ^31^P NMR readings were taken at 37°C. Two baseline readings were made: the first using ProTide **16** (5.0 mg) combined with DMSO‐d6 (0.10 mL) and D_2_O (0.15 mL), and the second using thawed human serum (0.3 mL), DMSO‐d6 (0.10 mL), and D_2_O (0.15 mL). After this, either human serum that had been previously thawed (0.30 mL) was introduced to the NMR tube, and a reading was taken right away. Subsequent readings were taken 30 min post addition and then at consistent 2 h intervals over a 12‐hour period.

### 4.15 Biological Assays

The methods are published in references 38,39. All biological assays were performed in at least three independent experiments (*n* = 3), each carried out in triplicate. Data are presented as the mean **±**standard deviation (SD). IC_50_ values were determined by non‐linear regression analysis using a four‐parameter logistic model (log(inhibitor) vs. response ‐‐ Variable slope) in GraphPad Prism software (GraphPad Software, San Diego, CA, USA).

### 4.16 Activity against Trypanosoma Brucei Rhodesiense STIB900.

This stock was isolated in 1982 from a human patient in Tanzania and after several mouse passages cloned and adapted to axenic culture conditions (Baltz et al. 1985) Minimum Essential Medium (50 µL) supplemented with 25 mM HEPES, 1 g/L additional glucose, 1% MEM nonessential amino acids (100x), 0.2 mM 2‐mercaptoethanol, 1 mM Na‐pyruvate, and 15% heat‐inactivated horse serum was added to each well of a 96‐well microtiter plate. Serial drug dilutions of eleven 3‐fold dilution steps covering a range from 100 to 0.002 μg/mL were prepared. Then 4 × 10^3^ bloodstream forms of *T. b. rhodesiense* STIB 900 in 50 µL was added to each well and the plate incubated at 37°C under a 5% CO_2_ atmosphere for 70 h. 10 µL resazurin solution (resazurin, 12.5 mg in 100 mL double‐distilled water) was then added to each well and incubation continued for a further 2–4 h (Raz et al. 1997). Then, the plates were read with a Spectramax Gemini XS microplate fluorometer (Molecular Devices Cooperation, Sunnyvale, CA, USA) using an excitation wavelength of 536 nm and an emission wavelength of 588 nm. Data were analyzed with the graphic programme Softmax Pro (Molecular Devices Cooperation, Sunnyvale, CA, USA), which calculated IC_50_ values by linear regression (Huber 1993) and 4‐parameter logistic regression from the sigmoidal dose inhibition curves. Mel (Arsobal Sanofi‐Aventis, received from WHO) is used as control.

### 4.17 Activity against T. cruzi

Rat skeletal myoblasts (L‐6 cells) were seeded in 96‐well microlitre plates at 2000 cells/well in 100 μL RPMI 1640 medium with 10% Fetal Bovine Serum (FBS) and 2 mM l‐glutamine. After 24 h the medium was removed and replaced by 100 μL per well containing 5000 trypomastigote forms of *T. cruzi* Tulahuen strain C2C4 containing the β‐galactosidase (Lac Z) gene (Buckner et al. 1996). After 48 h, the medium was removed from the wells and replaced by 100 μL fresh medium with or without a serial drug dilution of eleven 3‐fold dilution steps covering a range from 100 to 0.002 μg/mL. After 96 h of incubation, the plates were inspected under an inverted microscope to assure growth of the controls and sterility. Then, the substrate CPRG/Nonidet (50 μL) was added to all wells. A colour reaction developed within 2–6 h and could be read photometrically at 540 nm. Data were analyzed with the graphic programme Softmax Pro (Molecular Devices), which calculated IC_50_ values by linear regression (Huber1993) and four‐parameter logistic regression from the sigmoidal dose inhibition curves. BZN is used as control (IC50 0.5 ± 0.2 µg/ml).

### 4.18 Activity against L. Donovani Axenic Amastigotes.

Amastigotes of *L. donovani* strain MHOM/ET/67/L82 are grown in axenic culture at 37°C in SM medium (Cunnigham et al. 1977) at pH 5.4 supplemented with 10% heat‐inactivated fetal bovine serum under an atmosphere of 5% CO_2_ in air. One hundred microlitres of culture medium with 10^5^ amastigotes from axenic culture with or without a serial drug dilution are seeded in 96‐well microlitre plates. Serial drug dilutions of eleven 3‐fold dilution steps covering a range from 100 to 0.002 μg/mL are prepared. After 70 h of incubation, the plates are inspected under an inverted microscope to assure growth of the controls and sterile conditions. 10 μL of resazurin (12.5 mg resazurin dissolved in 100 mL distilled water) are then added to each well, and the plates are incubated for another 2 h. Then, the plates are read with a Spectramax Gemini XS microplate fluorometer (Molecular Devices Cooperation, Sunnyvale, CA, USA) using an excitation wavelength of 536 nm and an emission wavelength of 588 nm. From the sigmoidal inhibition curves, the IC_50_ values are calculated by linear regression (Huber 1993) and four‐parameter logistic regression using SoftmaxPro software (Molecular Devices Cooperation, Sunnyvale, CA, USA).

### 4.19 In Vitro Cytotoxicity with L‐6 Cells.

Assays were performed in 96‐well microliter plates, each well containing 100 μL of RPMI 1640 medium supplemented with 1% L‐glutamine (200 mM) and 10% fetal bovine serum, and 4000 L‐6 cells (rat skeletal myoblasts; RRID:CVCL_0385; ATCC CRL‐1458) (Page et al., 1993 and Ahmed et al., 1994). Serial drug dilutions of eleven 3‐fold dilution steps covering a range from 100 to 0.002 μg/mL were prepared. After 70 h of incubation, the plates were inspected under an inverted microscope to assure growth of the controls and sterile conditions. 10 μL of resazurin was then added to each well and the plates were incubated for another 2 h. Then, the plates were read with a Spectramax Gemini XS microplate fluorometer (Molecular Devices Cooperation, Sunnyvale, CA, USA) using an excitation wavelength of 536 nm and an emission wavelength of 588 nm. The IC50 values were calculated by linear regression (Huber 1993) and four‐parameter logistic regression from the sigmoidal dose inhibition curves using SoftmaxPro software (Molecular Devices Cooperation, Sunnyvale, CA, USA). Podophyllotoxin (Sigma P4405) is used as control.

## Supporting Information

Additional supporting information can be found online in the Supporting Information section.

## Funding

This work was supported by the Global Challenges Research Fund (MR/P027989/1A) and Royal Society of Chemistry (R21‐6118257436).

## Conflicts of Interest

The authors declare no conflicts of interest.

## Supporting information

Supplementary Material

## Data Availability

The data that support the findings of this study are available in the supplementary material of this article.
