# Supplementary material for: Targeting Kinetoplastid Parasites with ProTide Prodrugs: A Proof‐of‐Concept Study
Source: ChemMedChem. 2026 Apr 3;21(7):e202501072. doi: 10.1002/cmdc.202501072 (PMC13048835; doi:10.1002/cmdc.202501072)

Supplementary information

Targeting Kinetoplastid Parasites with ProTide Prodrugs: A Proof-of-Concept Study

Silvester Lowe [a], Vishal Satikuvar [a], Tanjia M Syeda [a], Monica Cal [b], Pascal Mäser [b], Marcel Kaiser [b] and Hachemi Kadri*[a]

[a] *S. Lowe, V. Satikuvar, T. M. Syeda, Dr. H. Kadri*
Department of Pharmacy, School of Life Sciences, Pharmacy and Chemistry, Kingston University, Penrhyn Road, Kingston upon Thames, London KT1 2EE, UK.
E-mail: h.kadri@kingston.ac.uk
[b] *M. Cal, Prof. P. Mäser, Dr. M. Kaiser*
Department of Medical Parasitology and Infection Biology, Swiss Tropical and Public Health Institute (Swiss TPH), Allschwil, Switzerland, University of Basel, Basel, Switzerland.

I. ^{31}P, ^{1}H and ^{13}C Spectra

1. Compound 9 1
2. Compound 10 3
3. Compound 11 5
4. Compound 12 7
5. Compound 13 9
6. Compound 14 11
7. Compound 15 13
8. Compound 16 15

II. HRMS and HPLC Spectra

1, Compound 9 17
2. Compound 10 18
3. Compound 11 19
4. Compound 12 20
5. Compound 13 21
6. Compound 14 22
7. Compound 15 23
8. Compound 16 24

I. ^{31}P, ^{1}H and ^{13}C Spectra

Compound 9

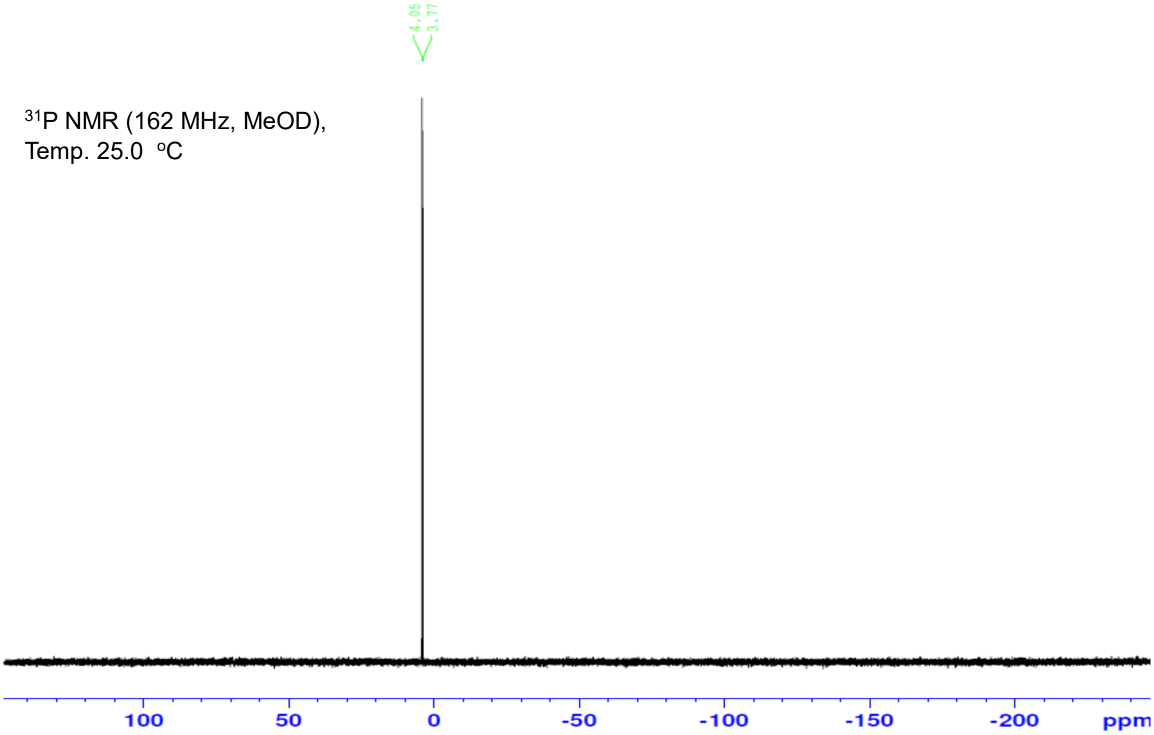

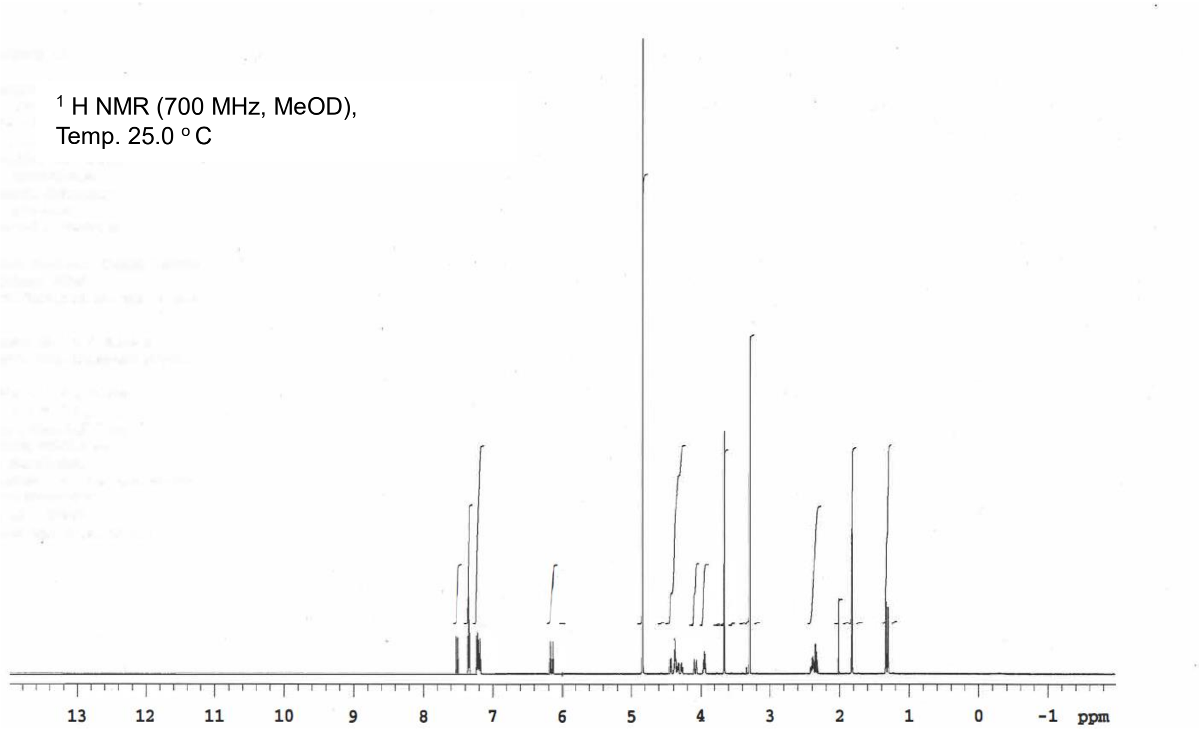

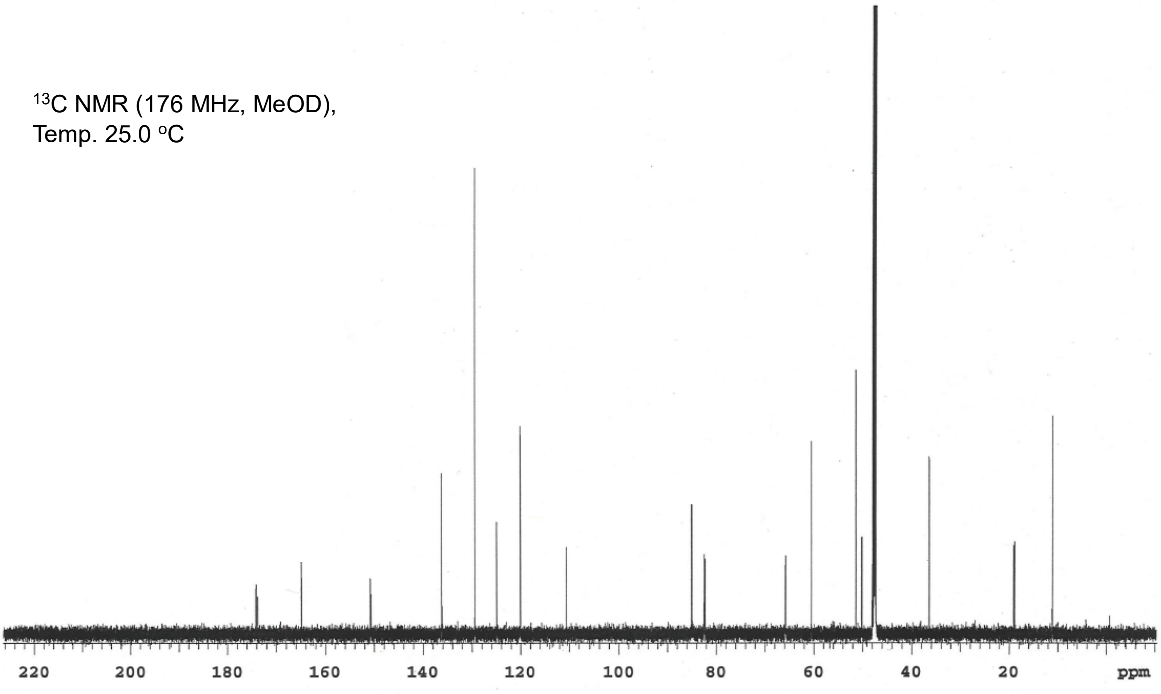
13C NMR (176 MHz, MeOD),
Temp. 25.0 oC
220
200
180
160
140
120
100
80
60
40
20
ppm

Compound 10

^{31}P NMR (162 MHz, MeOD),
Temp. 25.0 °C

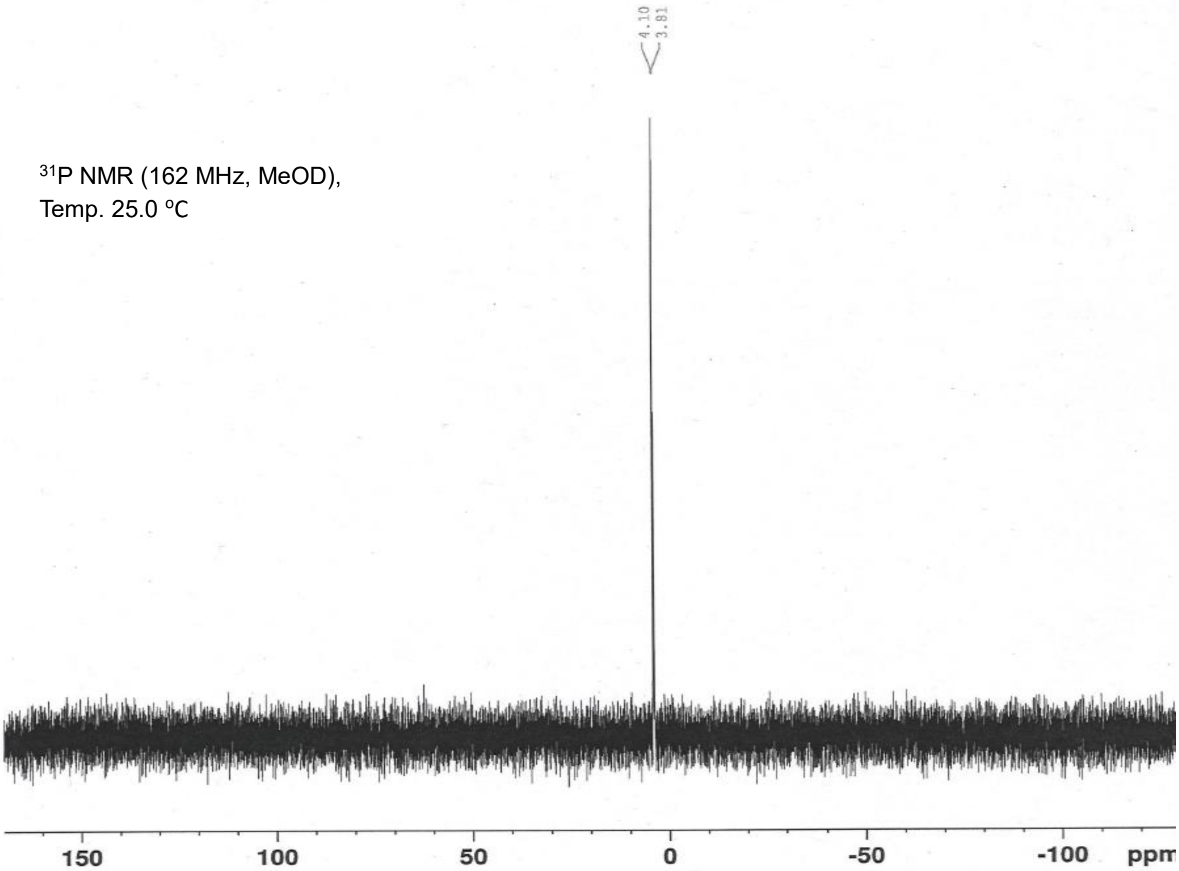

^{1}H NMR (700 MHz, MeOD),
Temp. 25.0 °C

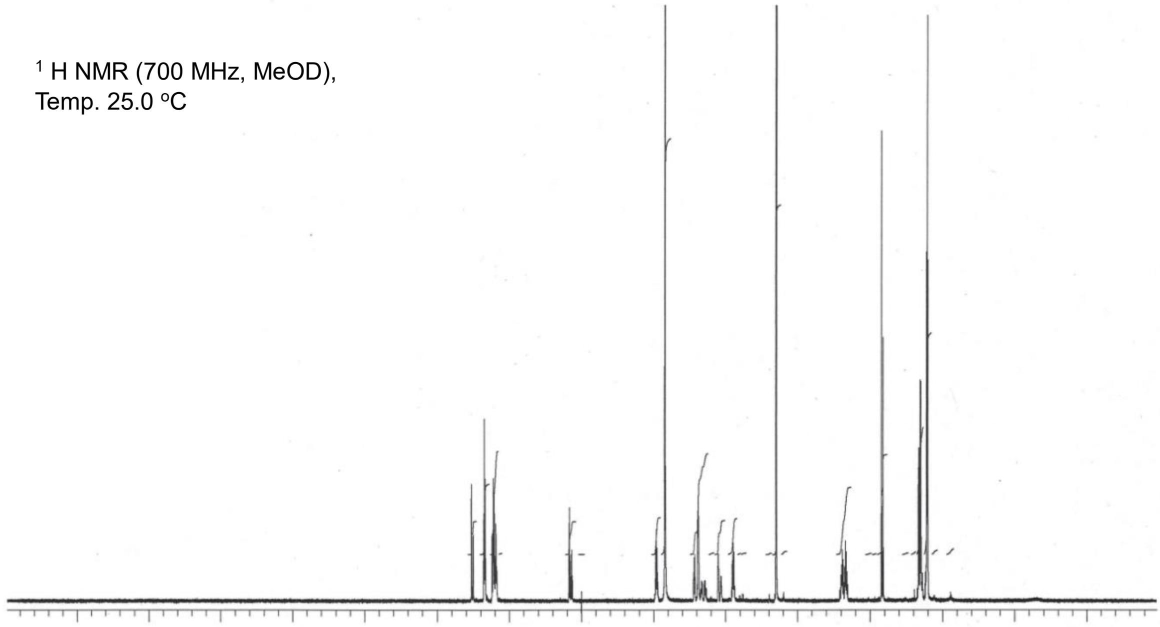

^{13}C NMR (176 MHz, MeOD),
Temp. 25.0 °C

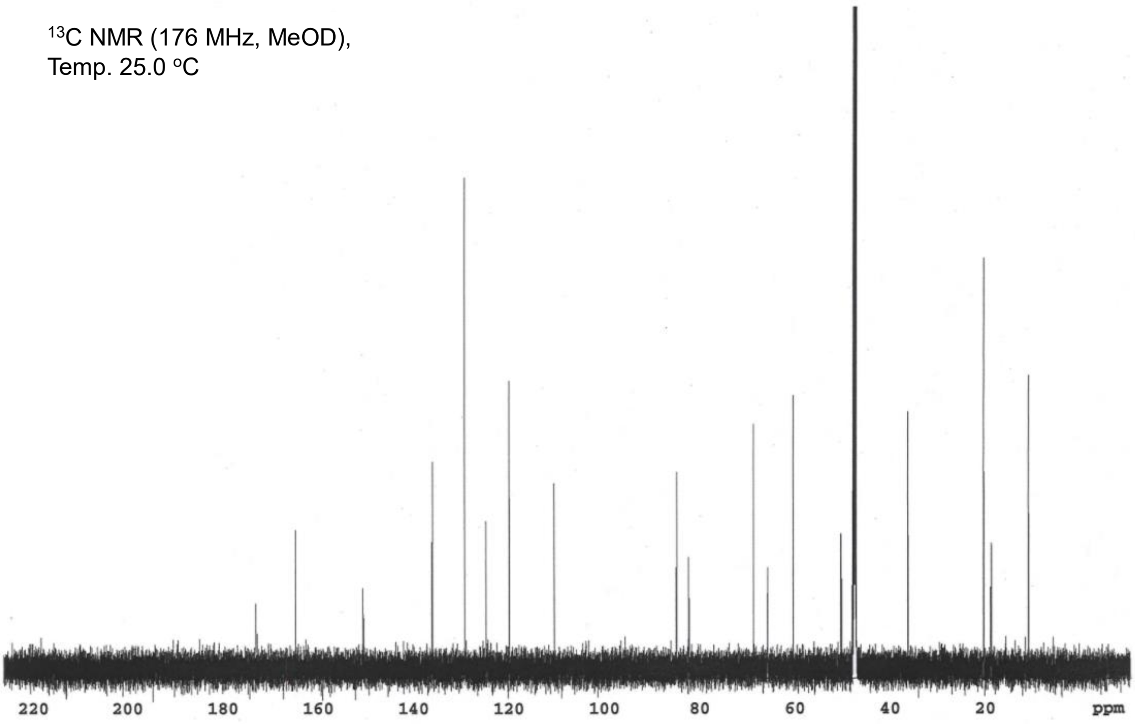

Compound 11

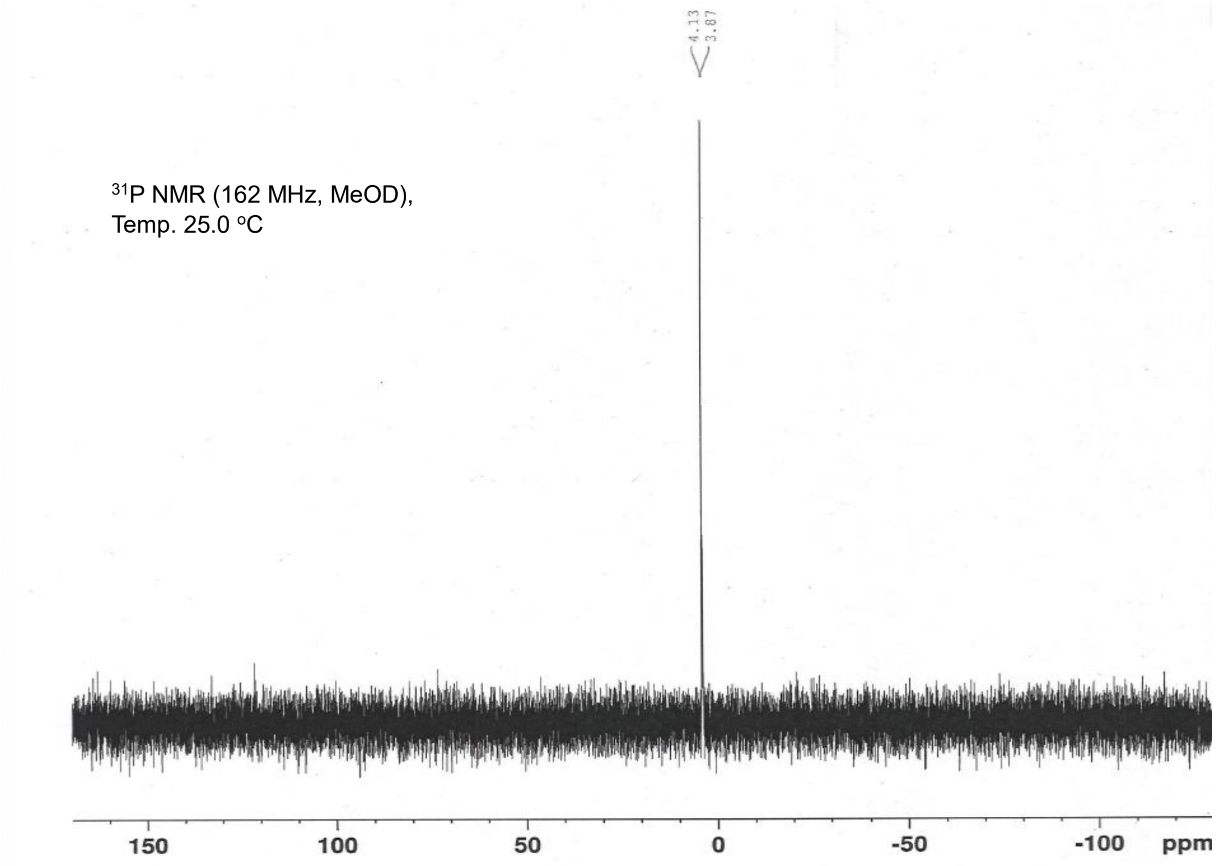
4.13
3.87
31P NMR (162 MHz, MeOD),
Temp. 25.0 ºC
150 100 50 0 -50 -100 ppm

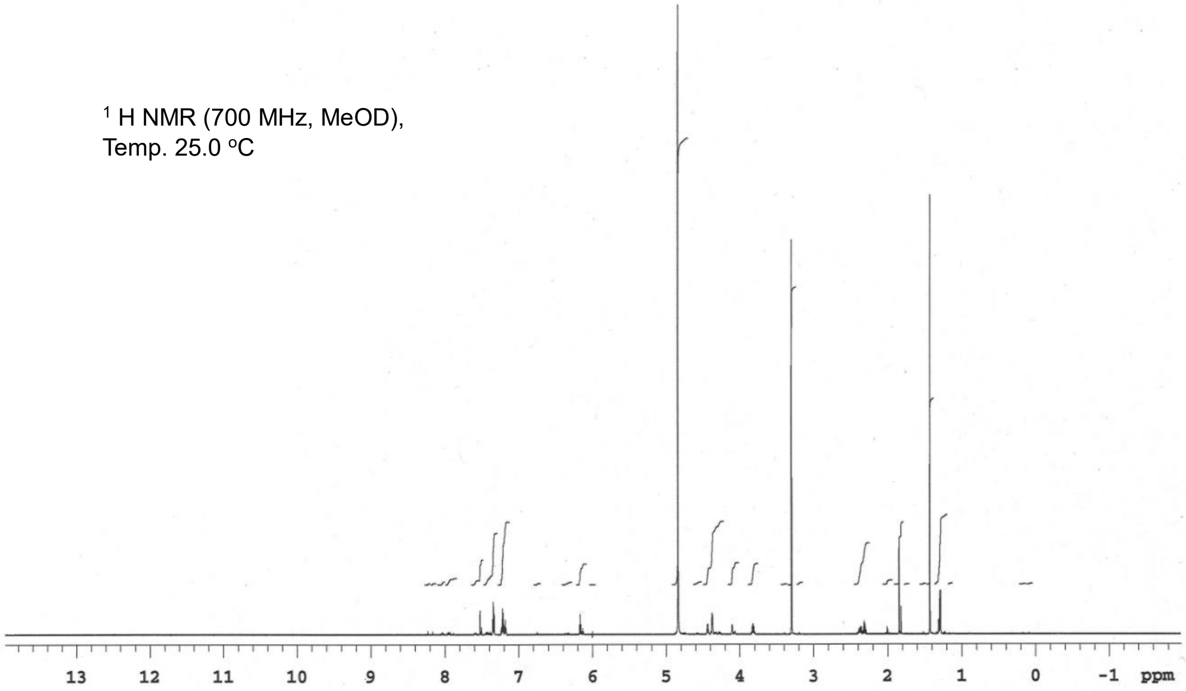
1 H NMR (700 MHz, MeOD),
Temp. 25.0 ºC
13 12 11 10 9 8 7 6 5 4 3 2 1 0 -1 ppm

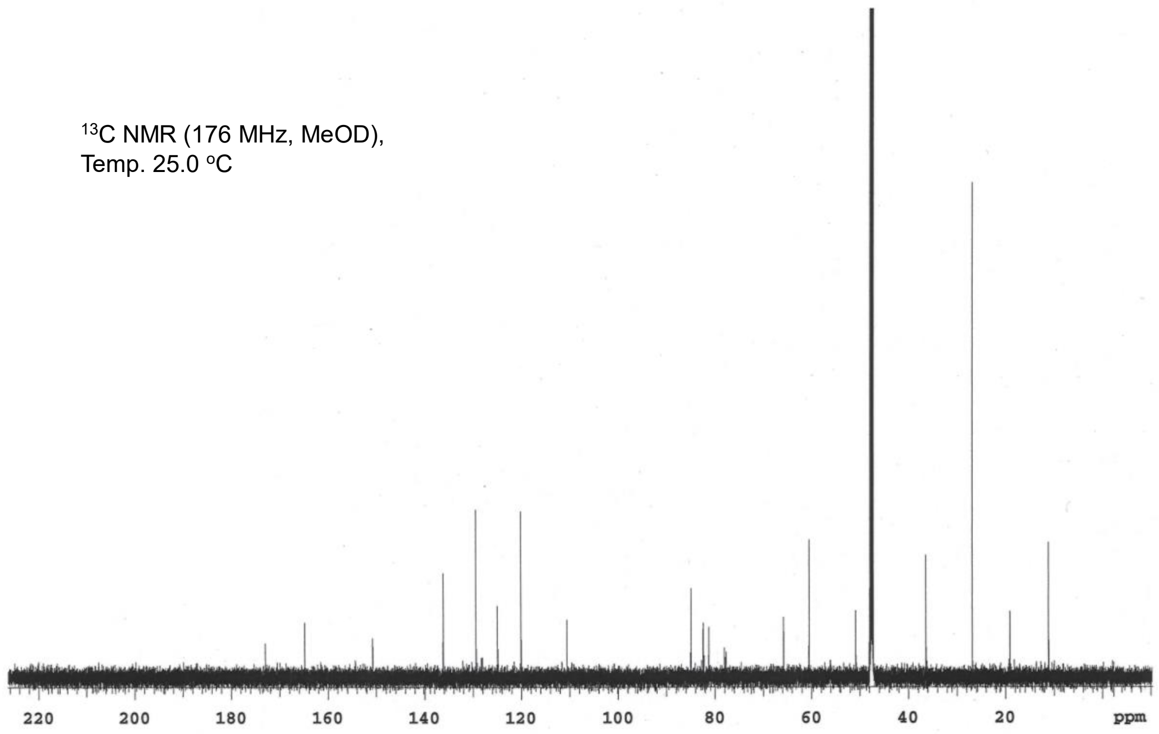
^{13}C NMR (176 MHz, MeOD),
Temp. 25.0 °C
220
200
180
160
140
120
100
80
60
40
20
ppm

Compound 12

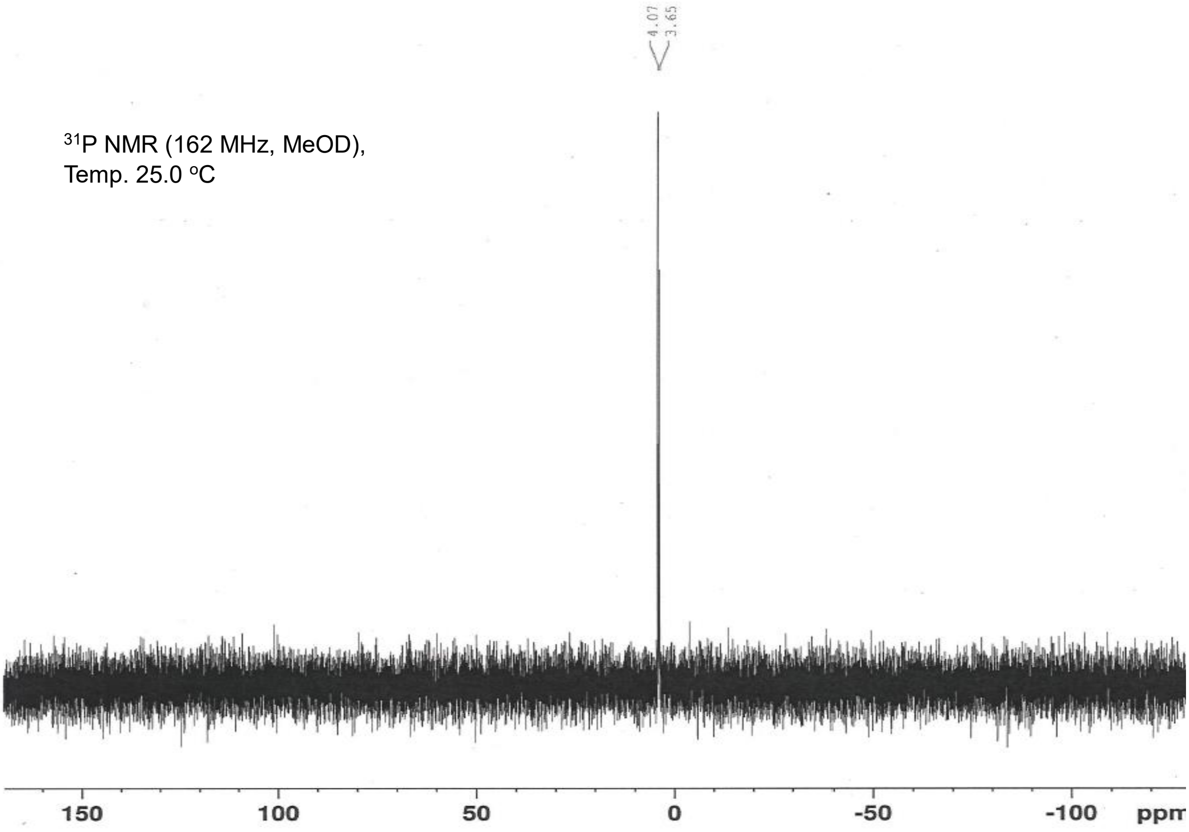

[1] H NMR (700 MHz, MeOD),
Temp. 25.0 °C

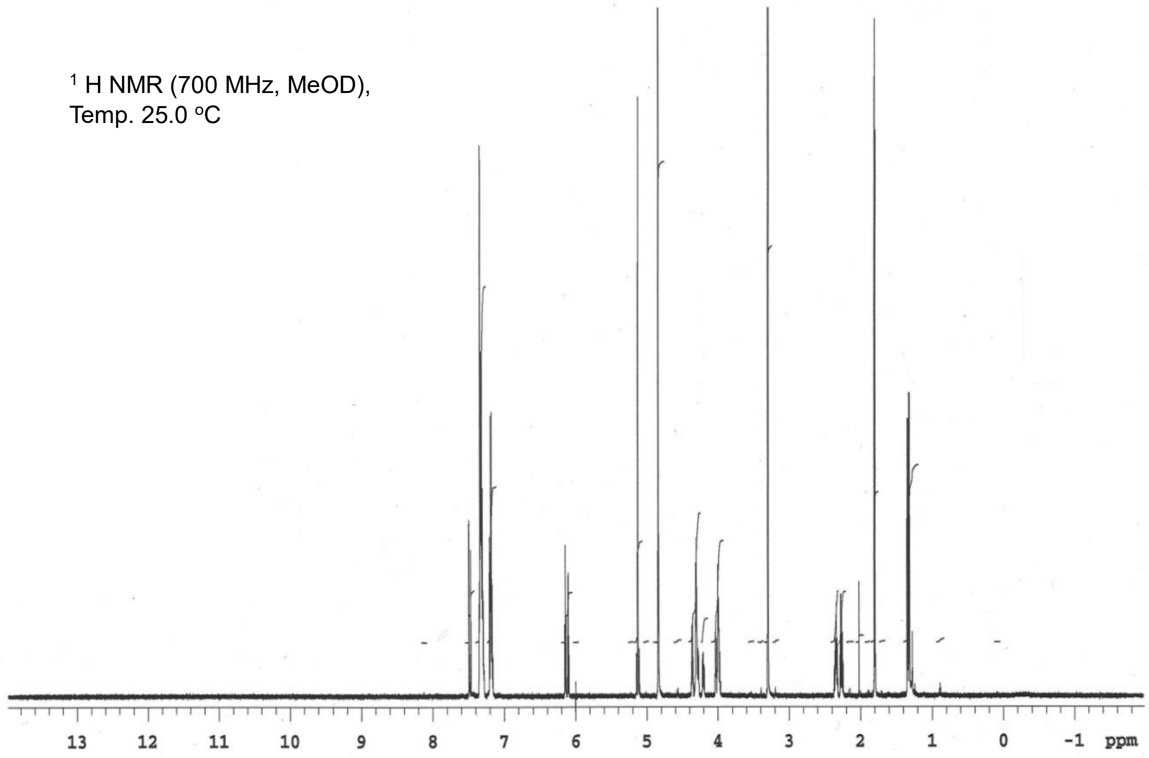

^{13}C NMR (176 MHz, MeOD),
Temp. 25.0 °C

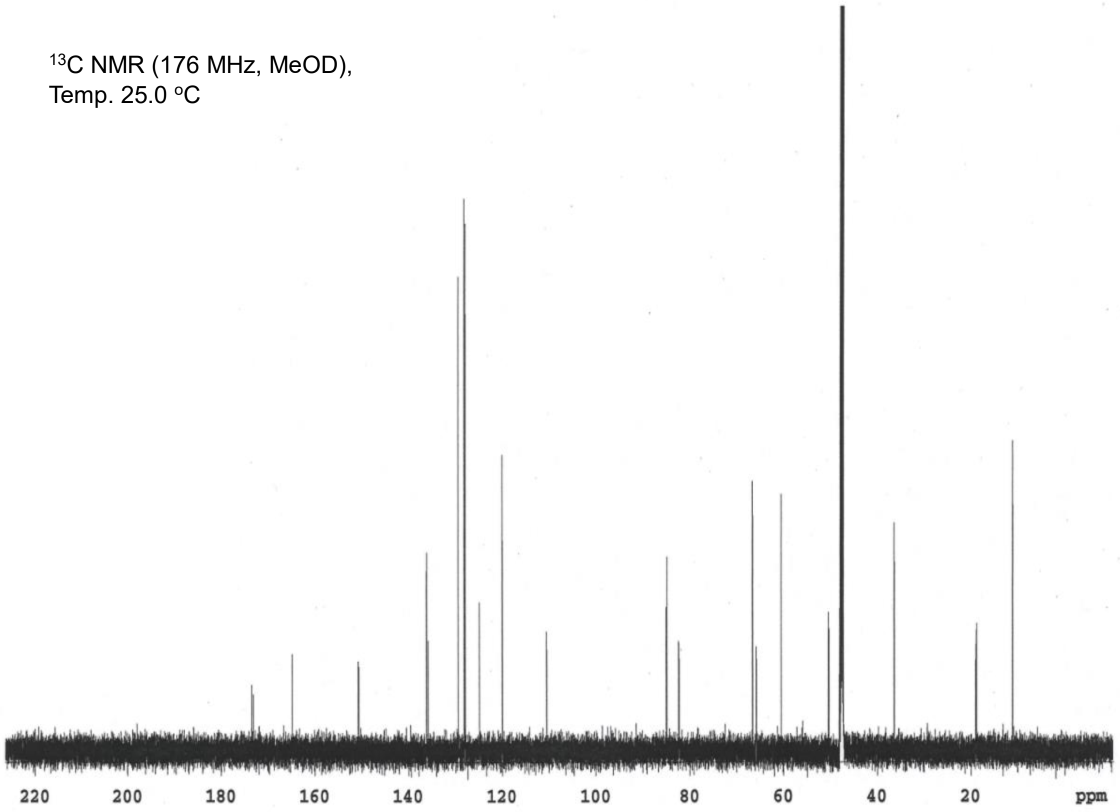

Compound 13

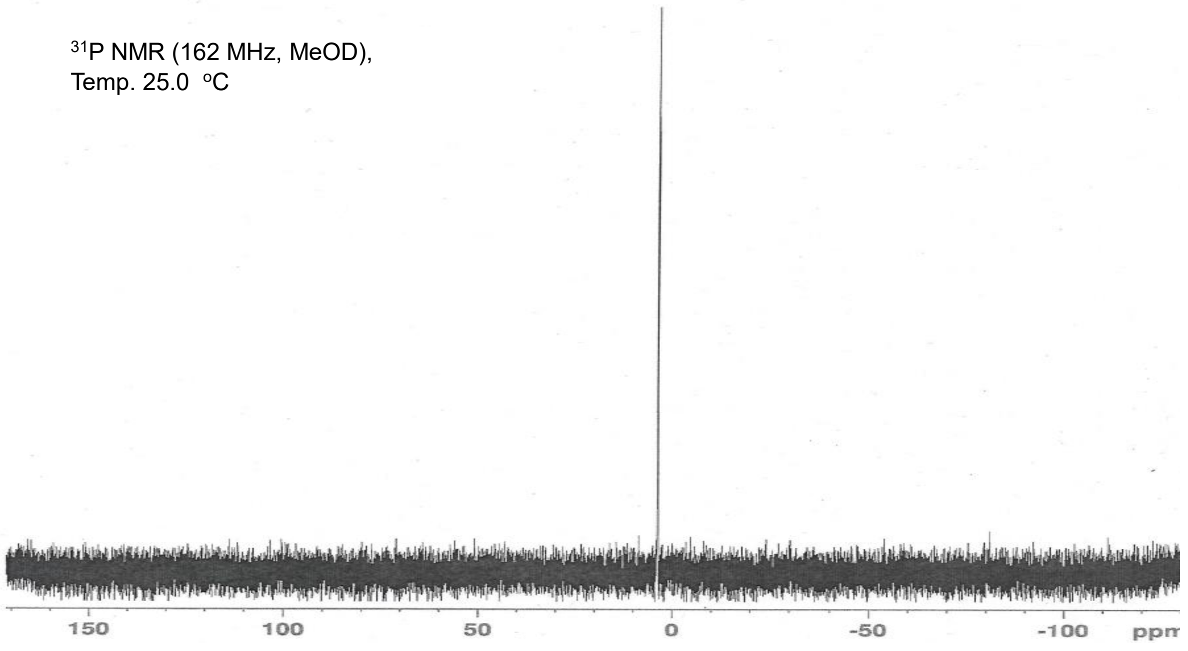

^{31}P NMR (162 MHz, MeOD),
Temp. 25.0 °C
150
100
50
0
-50
-100
ppm

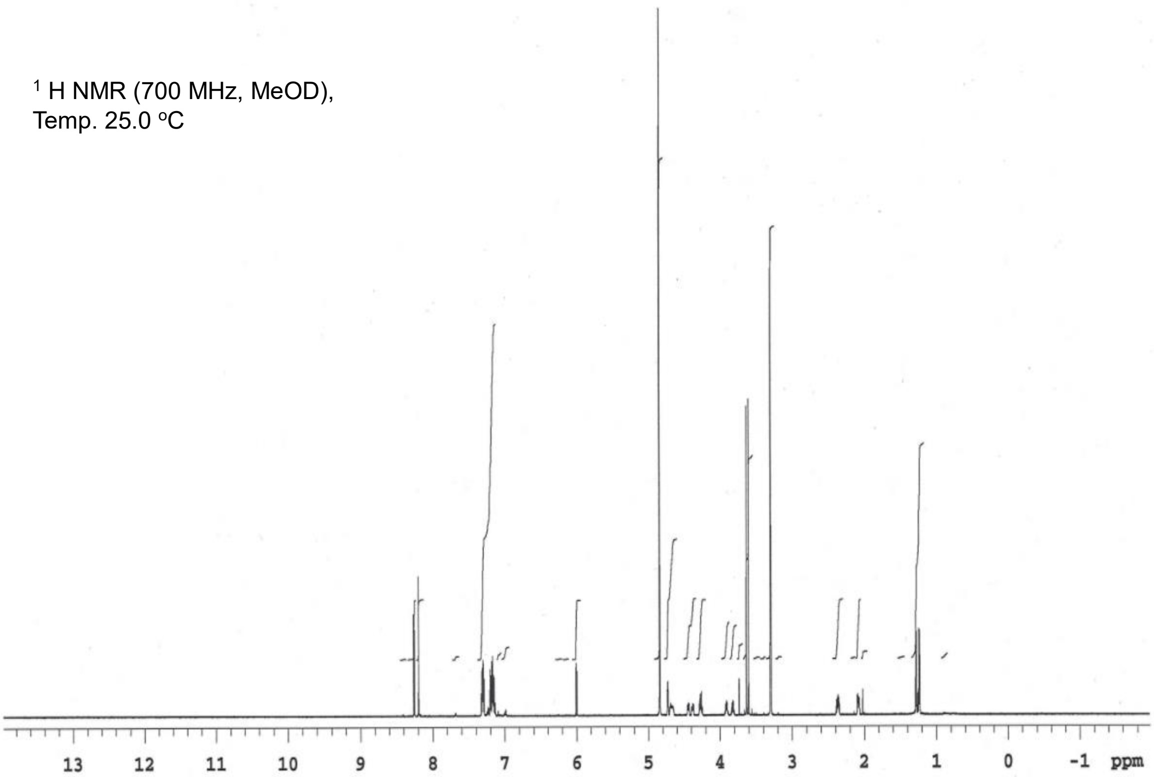

^{1}H NMR (700 MHz, MeOD),
Temp. 25.0 °C
13
12
11
10
9
8
7
6
5
4
3
2
1
0
-1
ppm

^{13}C NMR (176 MHz, MeOD),
Temp. 25.0 °C

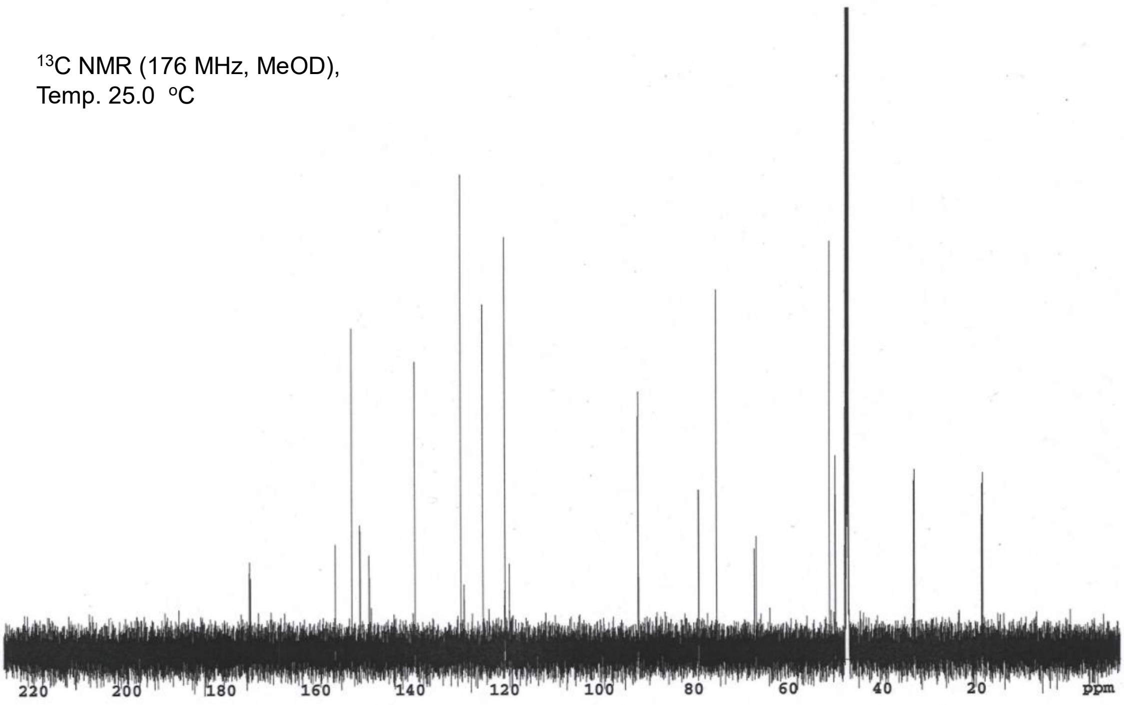

Compound 14

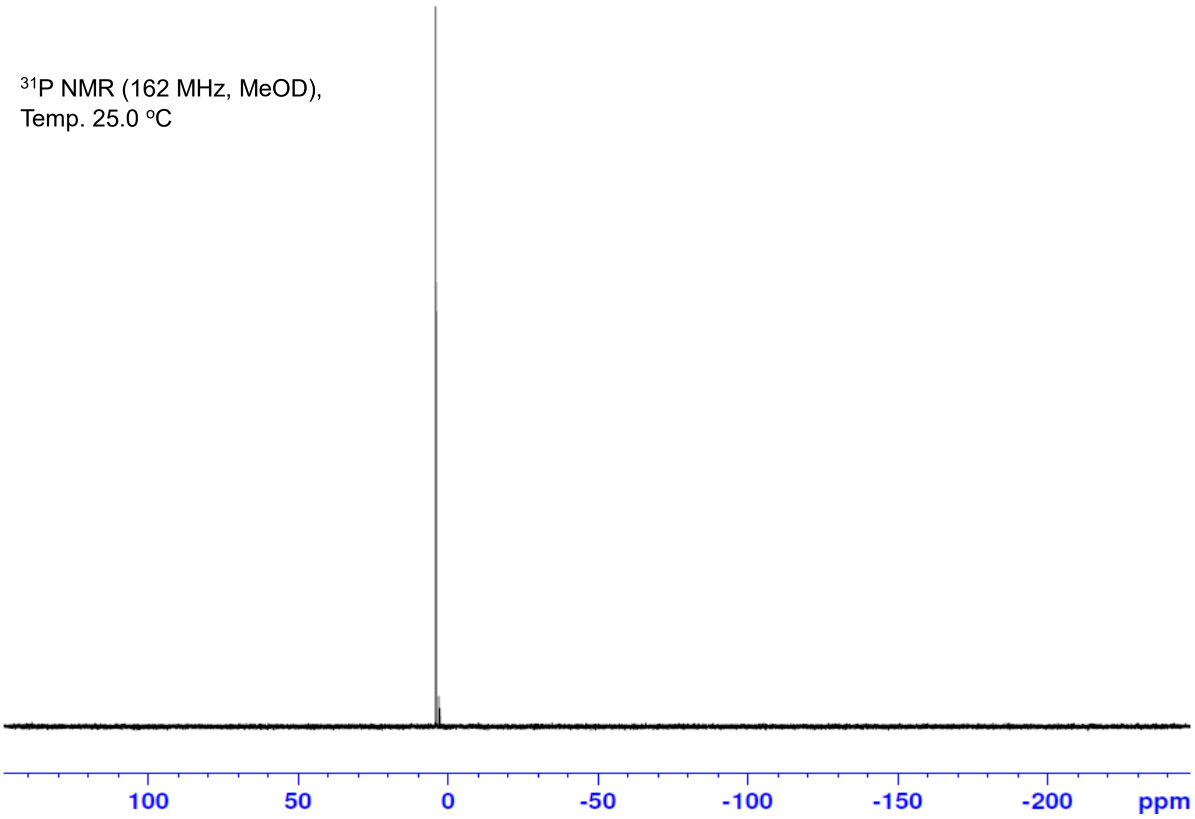
^{31}P NMR (162 MHz, MeOD),
Temp. 25.0 °C
100
50
0
-50
-100
-150
-200
ppm

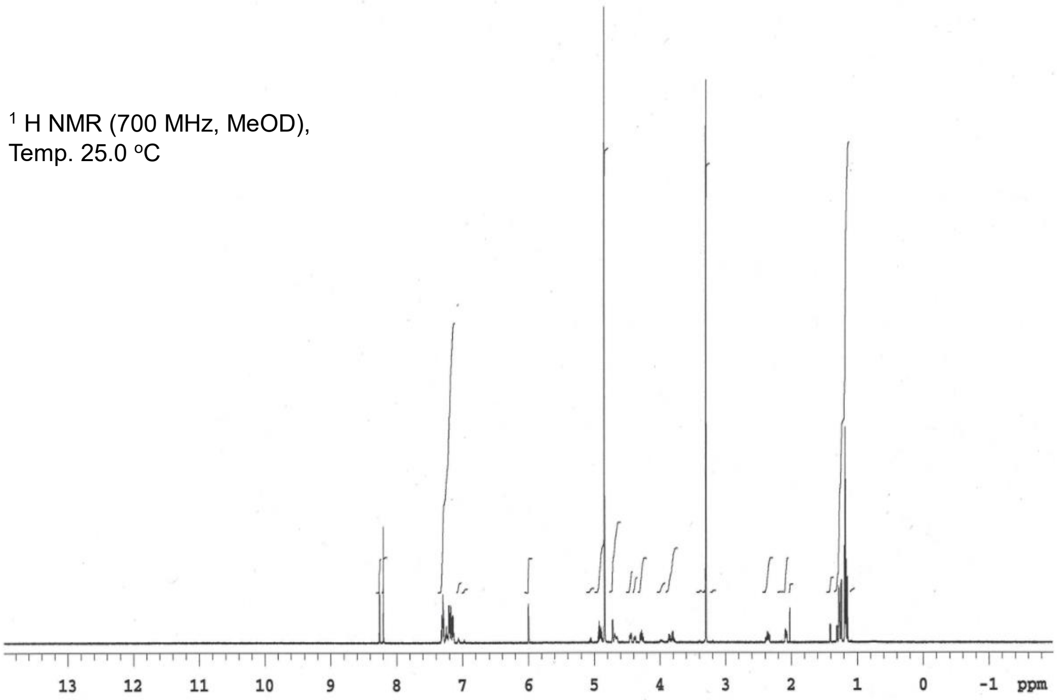
^{1}H NMR (700 MHz, MeOD),
Temp. 25.0 °C
13
12
11
10
9
8
7
6
5
4
3
2
1
0
-1
ppm

^{13}C NMR (176 MHz, MeOD),
Temp. 25.0 °C

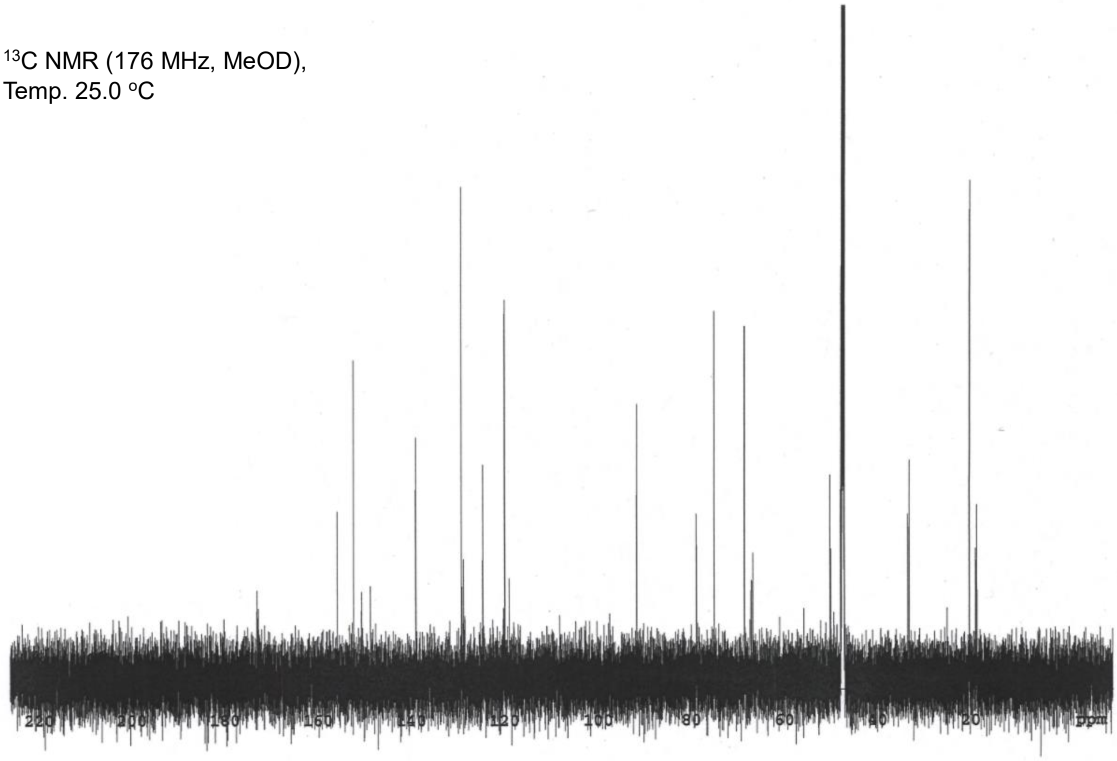

Compound 15

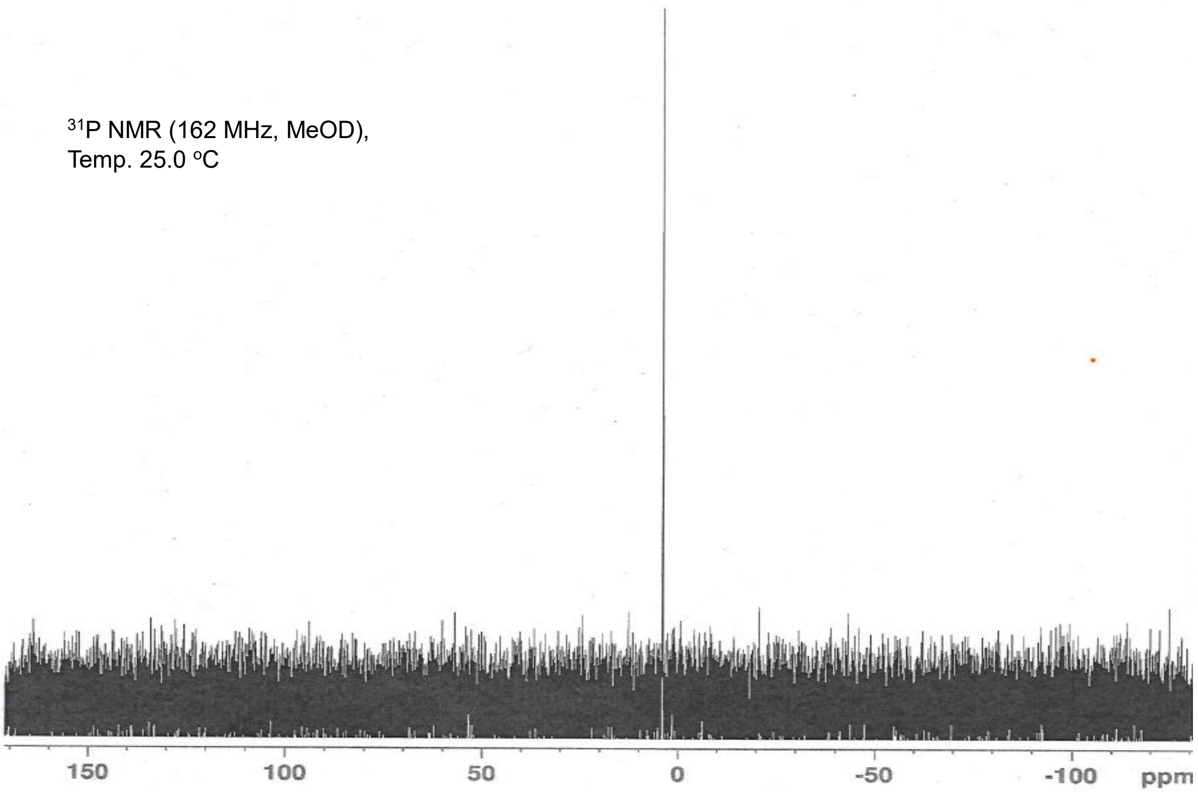

^{31}P NMR (162 MHz, MeOD),
Temp. 25.0 °C
150
100
50
0
-50
-100
ppm

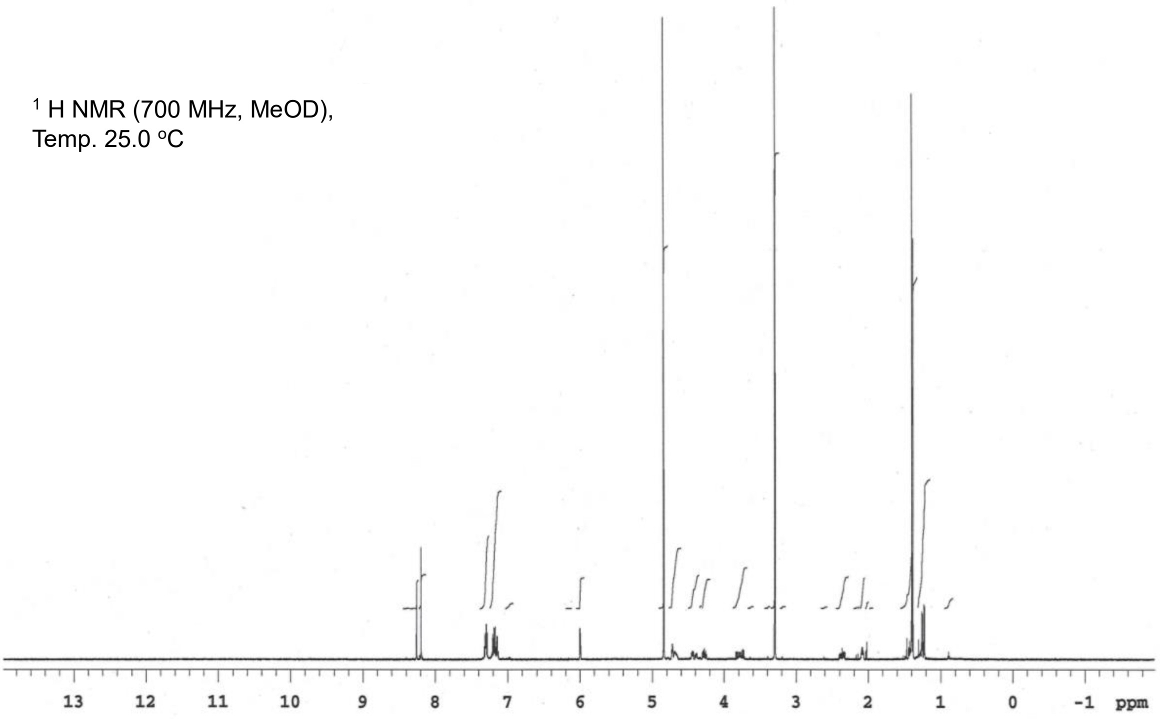

^{1} H NMR (700 MHz, MeOD),
Temp. 25.0 °C
13
12
11
10
9
8
7
6
5
4
3
2
1
0
-1
ppm

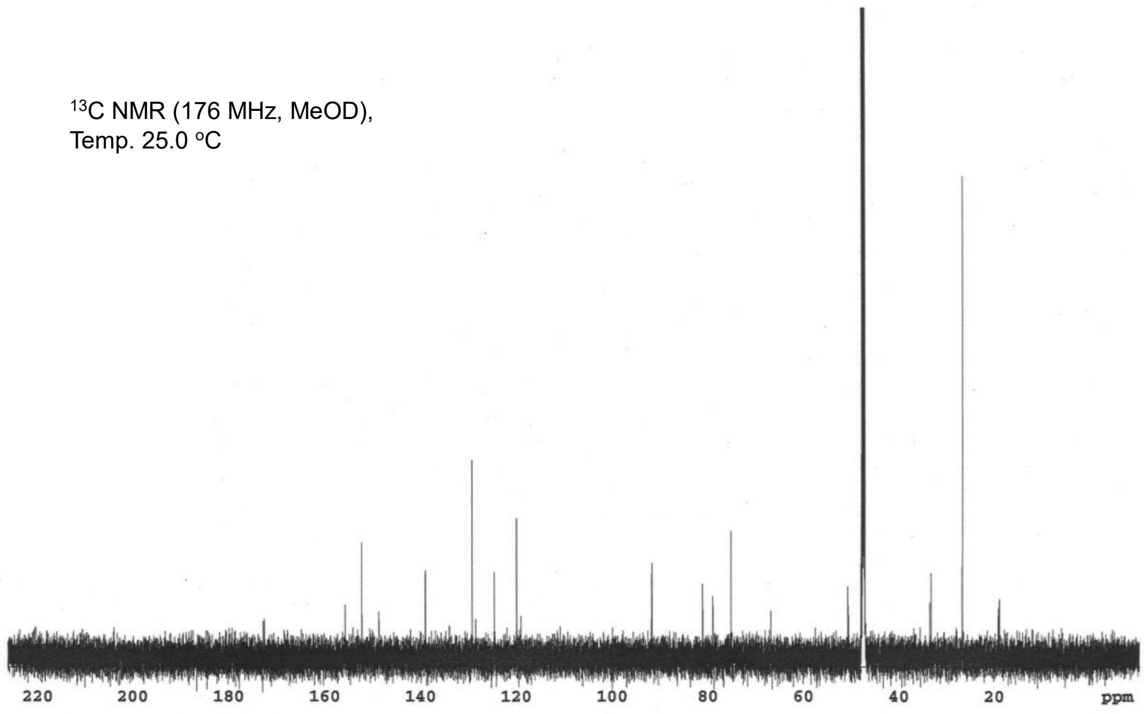
13C NMR (176 MHz, MeOD),
Temp. 25.0 oC
220
200
180
160
140
120
100
80
60
40
20
ppm

Compound 16

^{31}P NMR (162 MHz, MeOD),
Temp. 25.0 ^{o}C

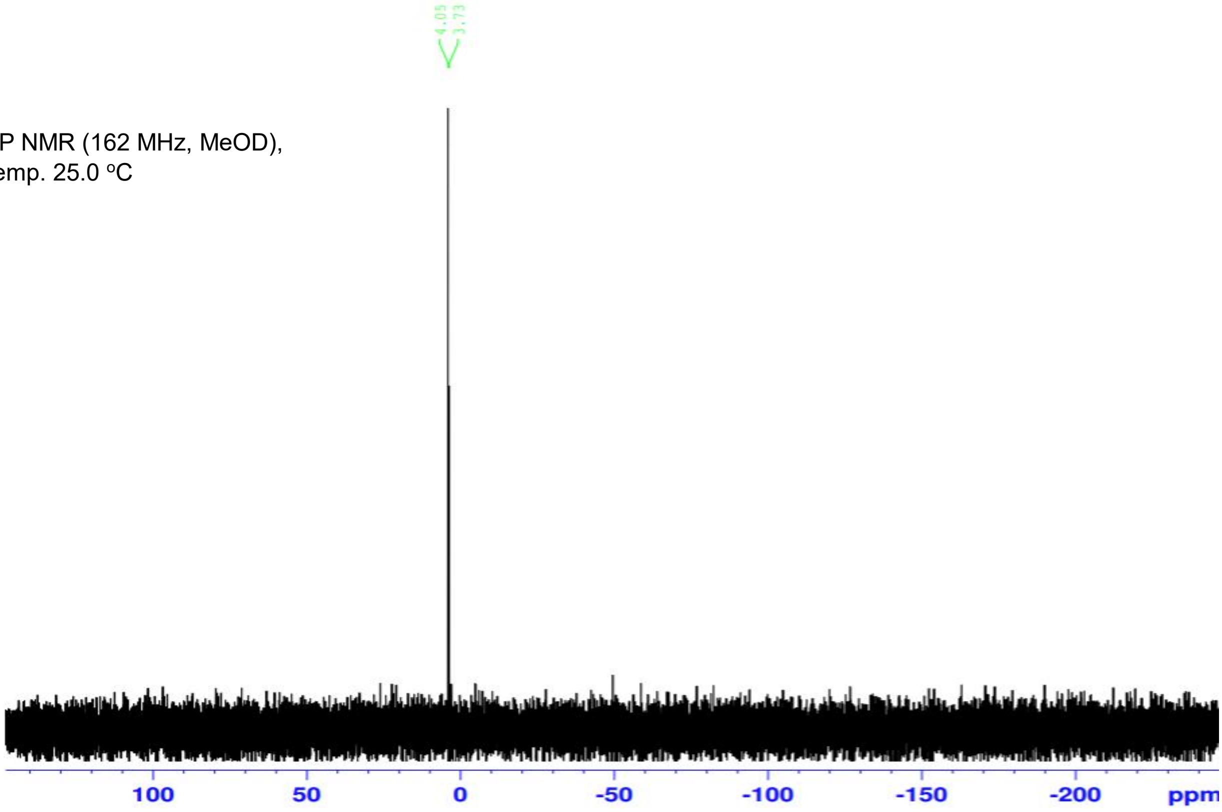

^{1} H NMR (600 MHz,
MeOD),
Temp. 25.0 ^{o}C

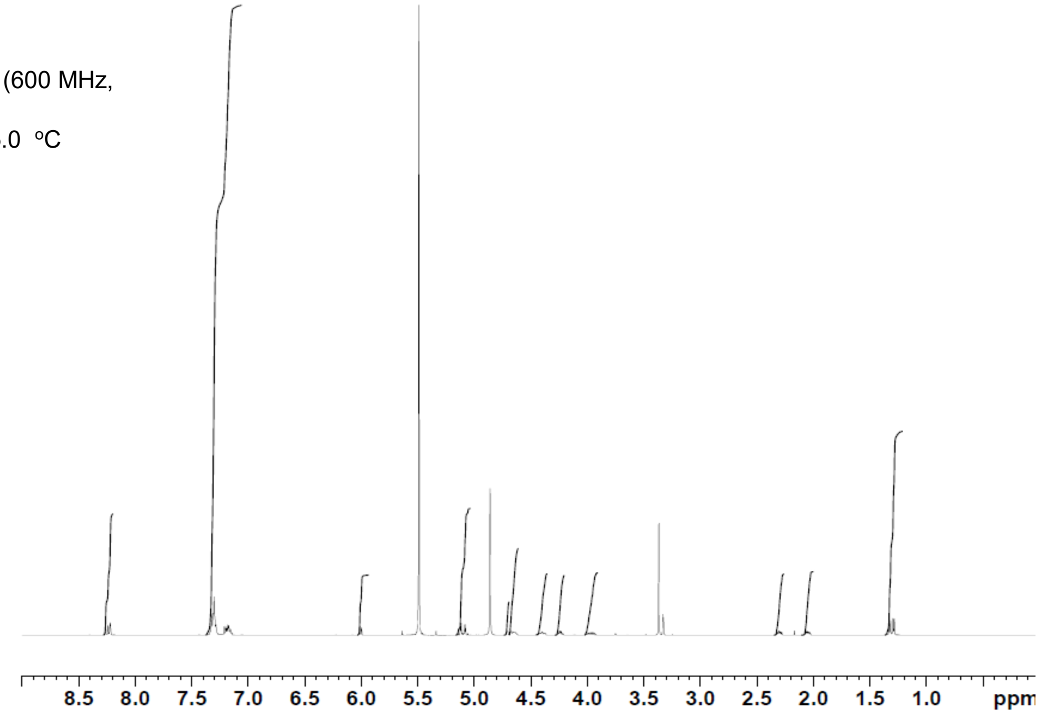

^{13}C NMR (176 MHz, MeOD),
Temp. 25.0 °C

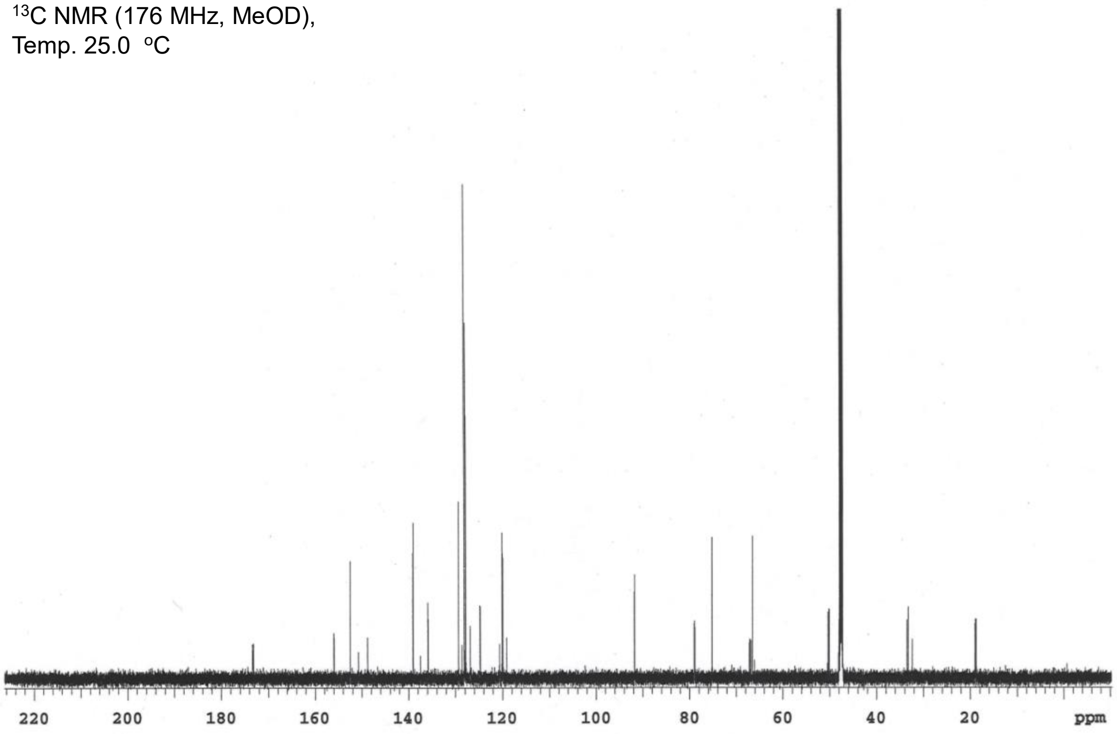

II. HRMS & HPLC

The HRMS spectrum of compound 9

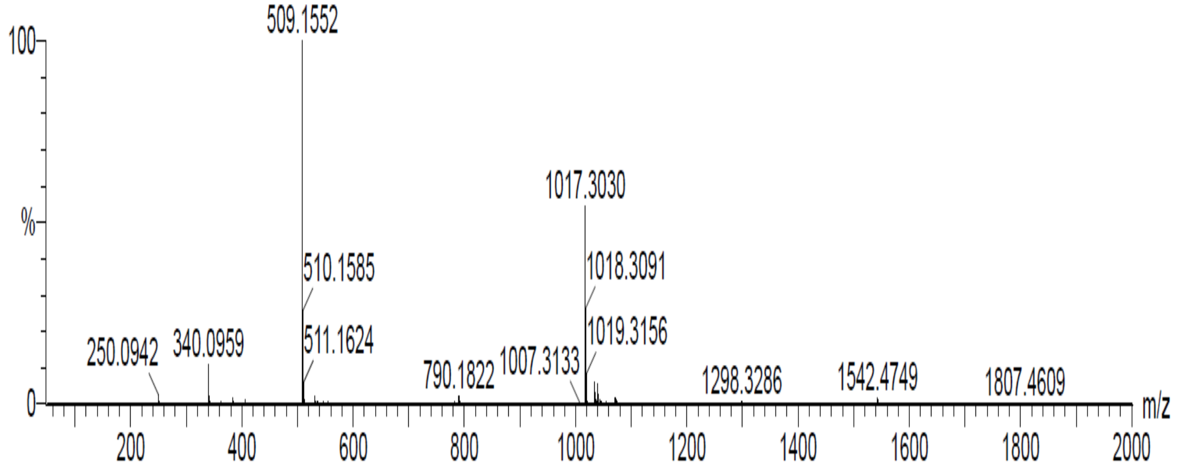

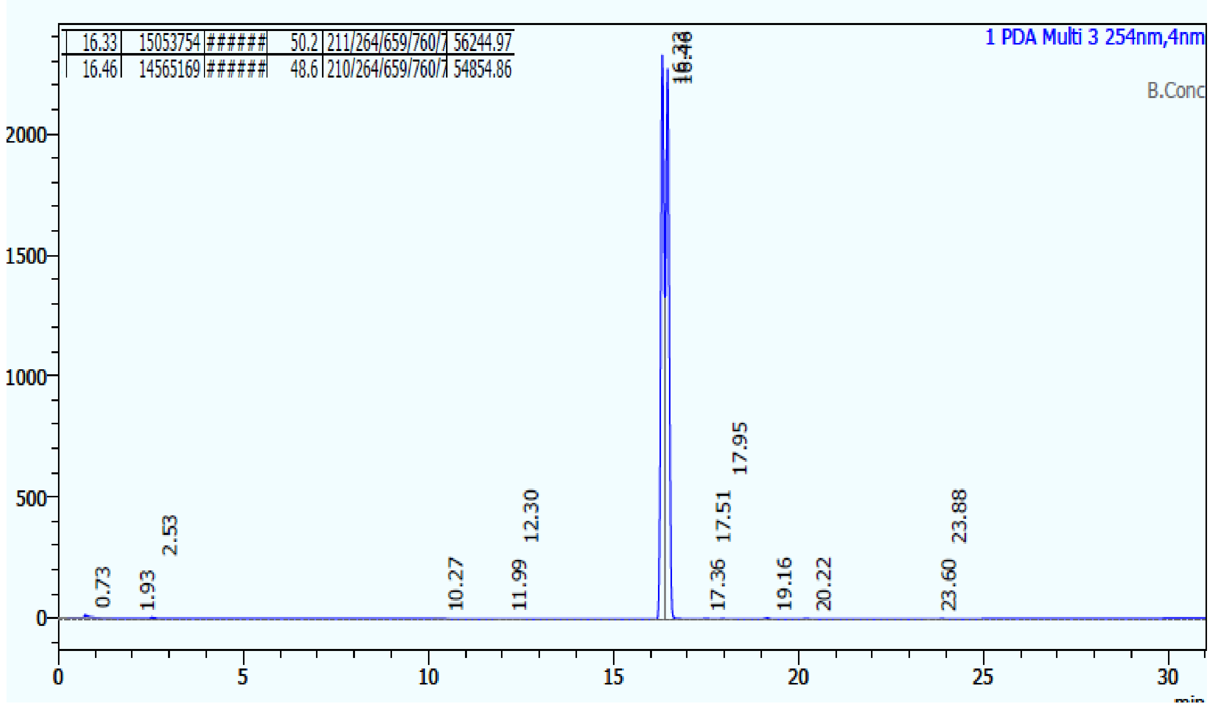

The HRMS spectrum of compound 10

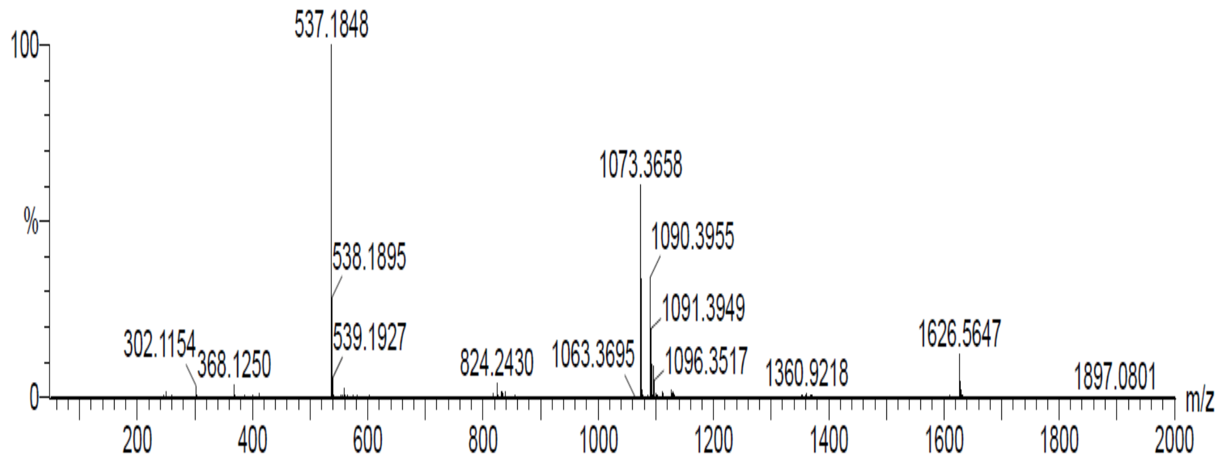

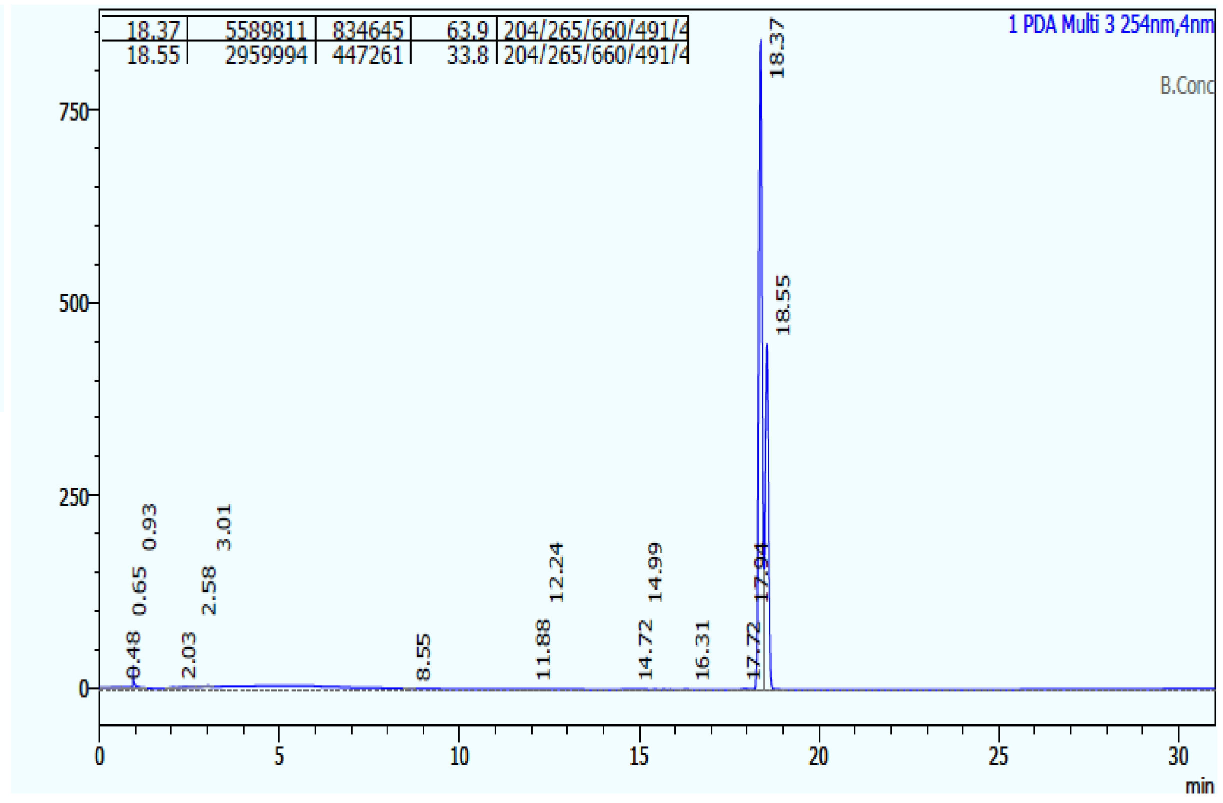

The HRMS spectrum of compound 11

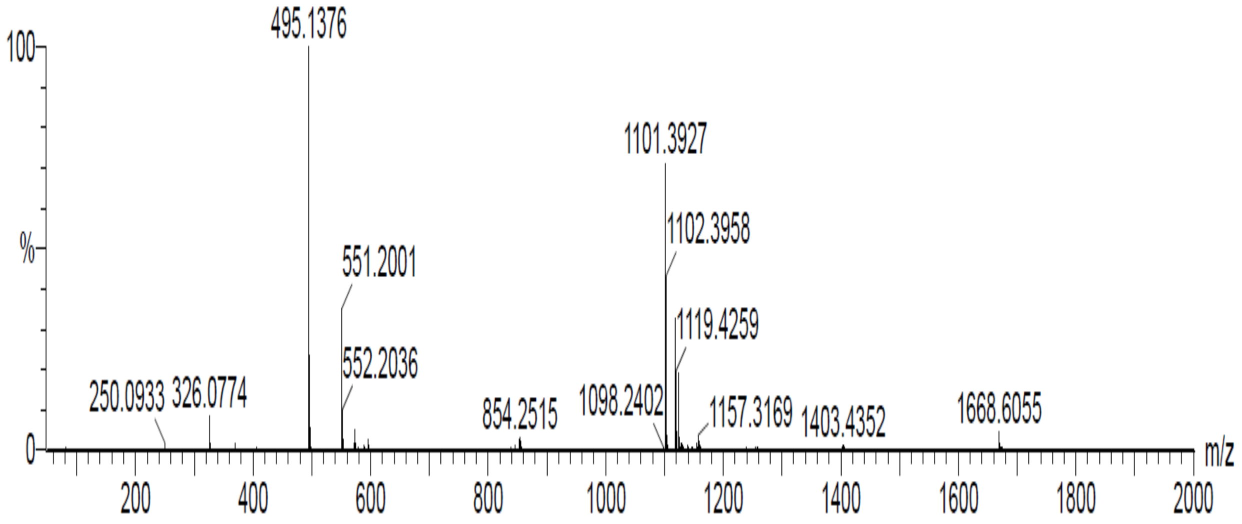

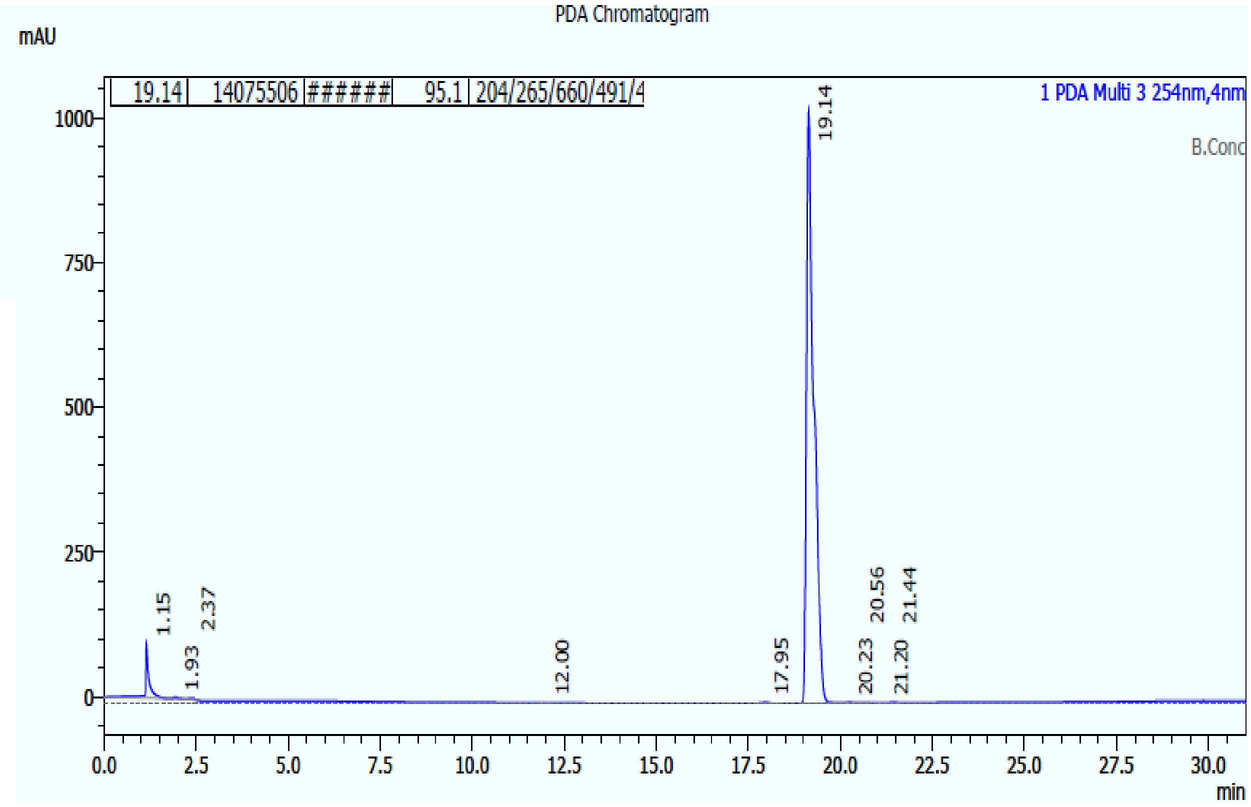

The HRMS spectrum of compound 12

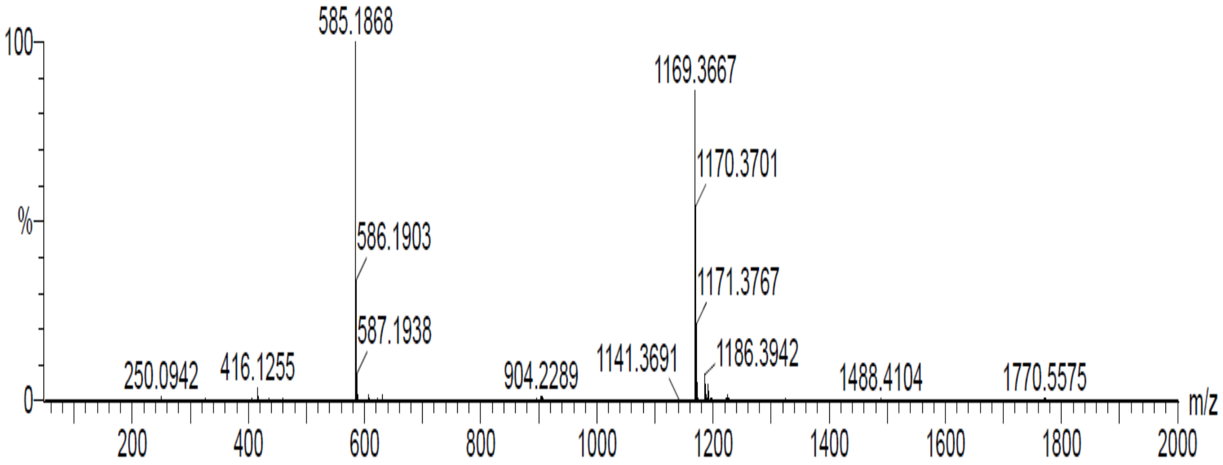

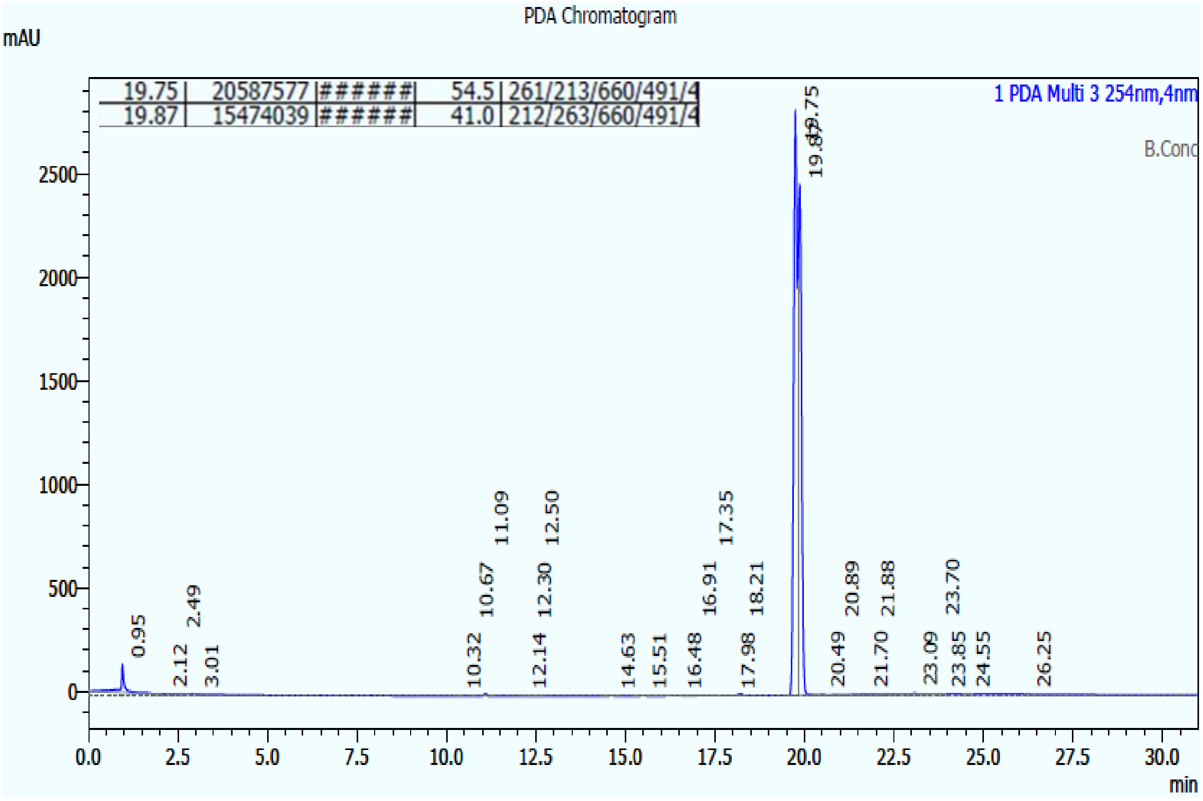

The HRMS spectrum of compound 13

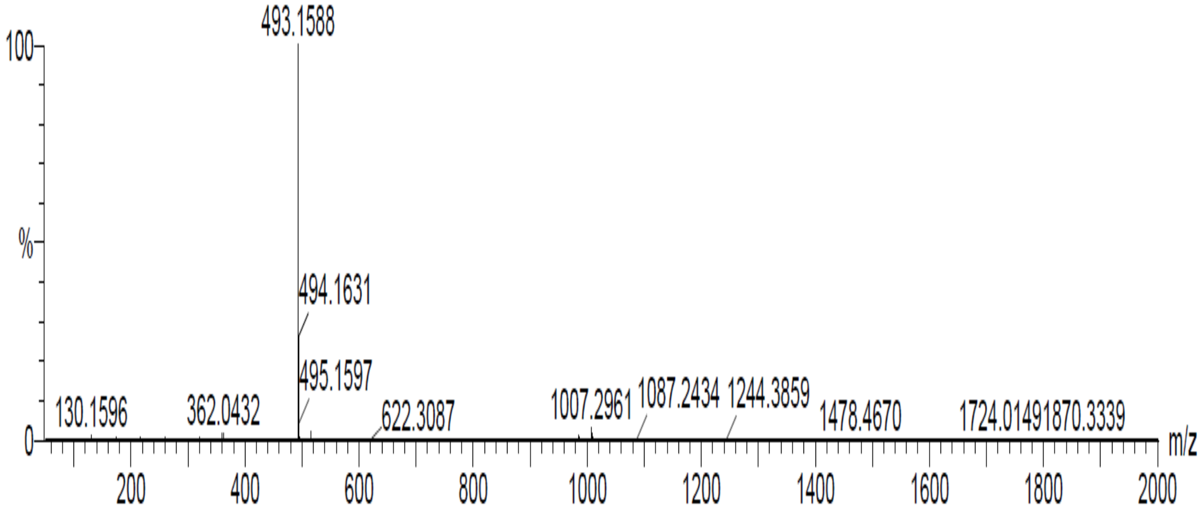

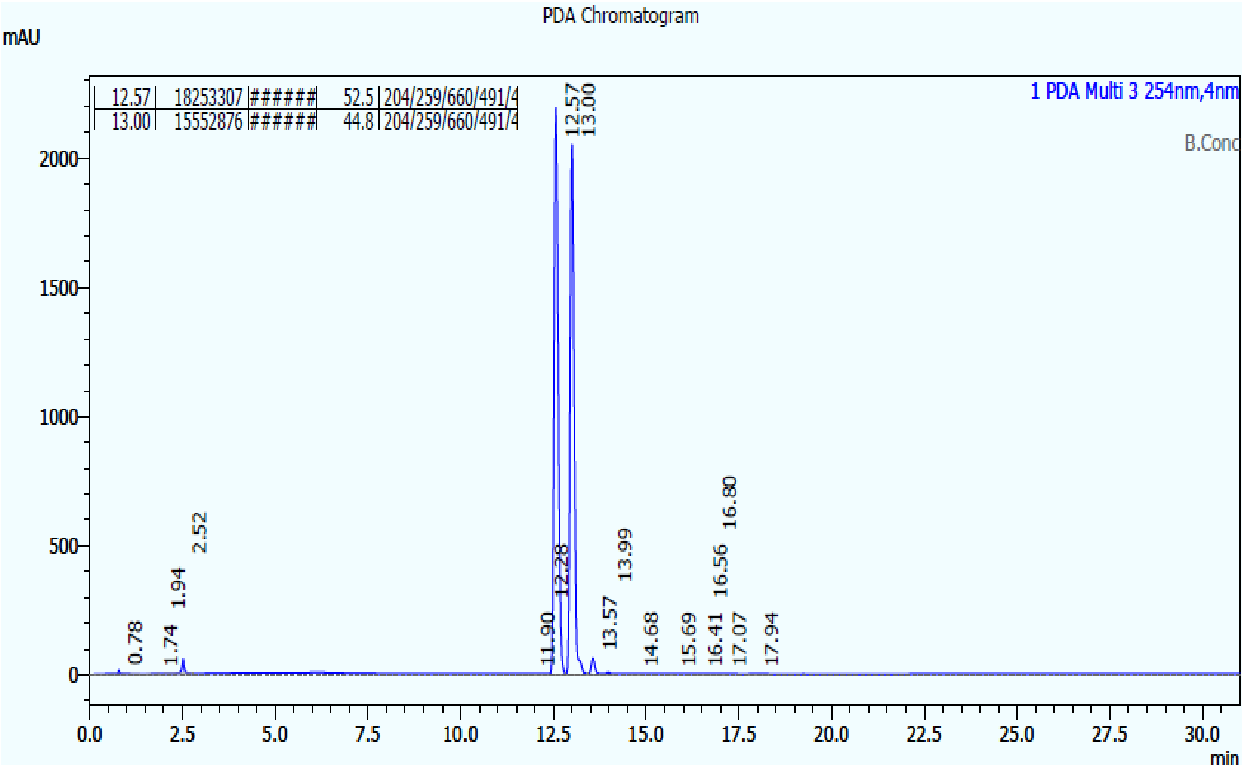

The HRMS spectrum of compound 14

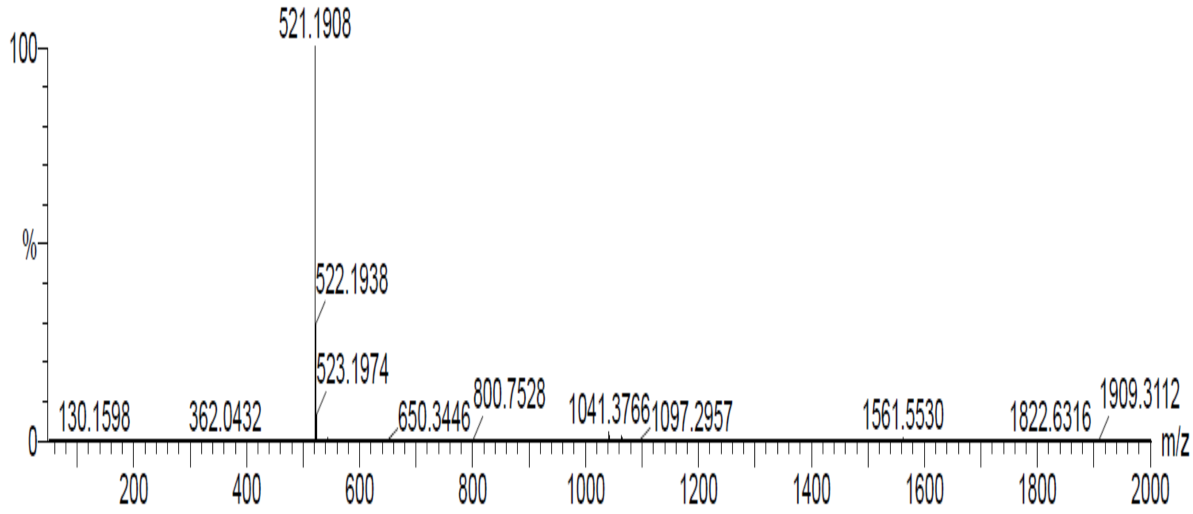

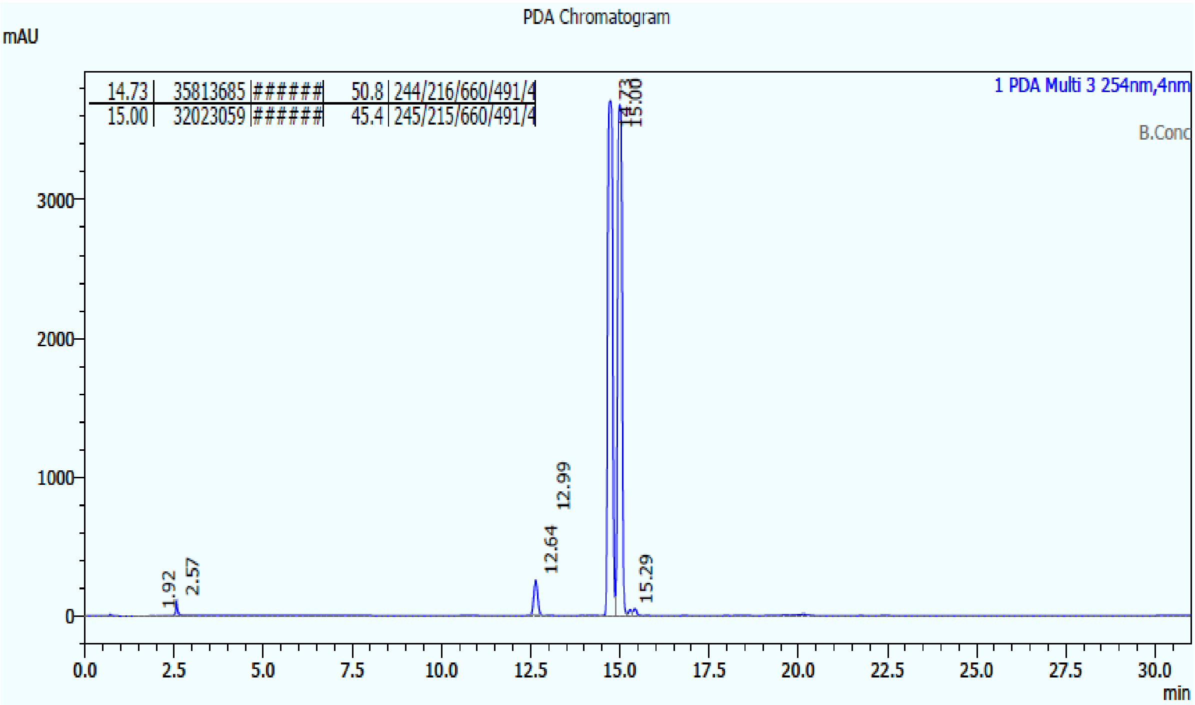

The HRMS spectrum of compound 15

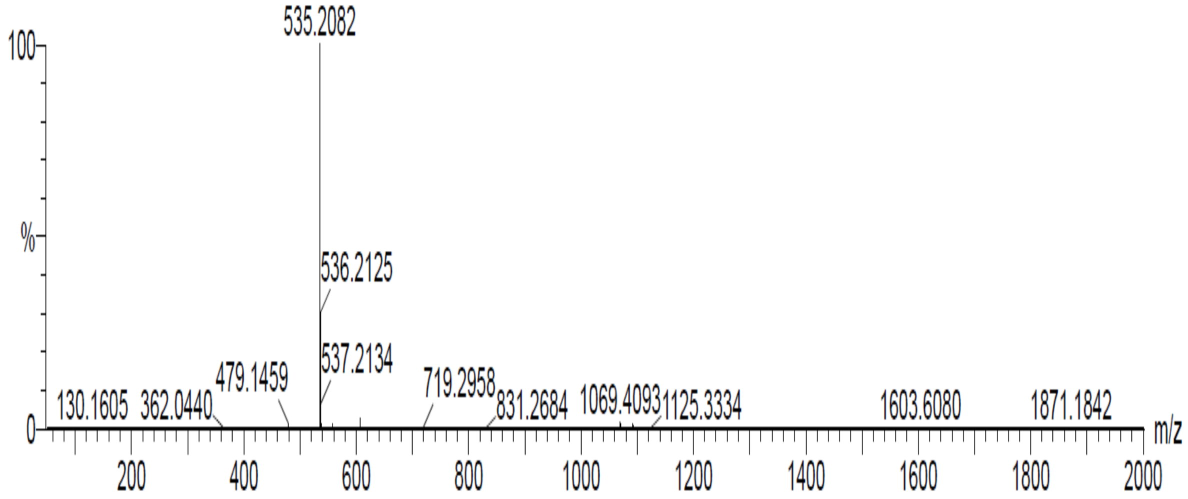

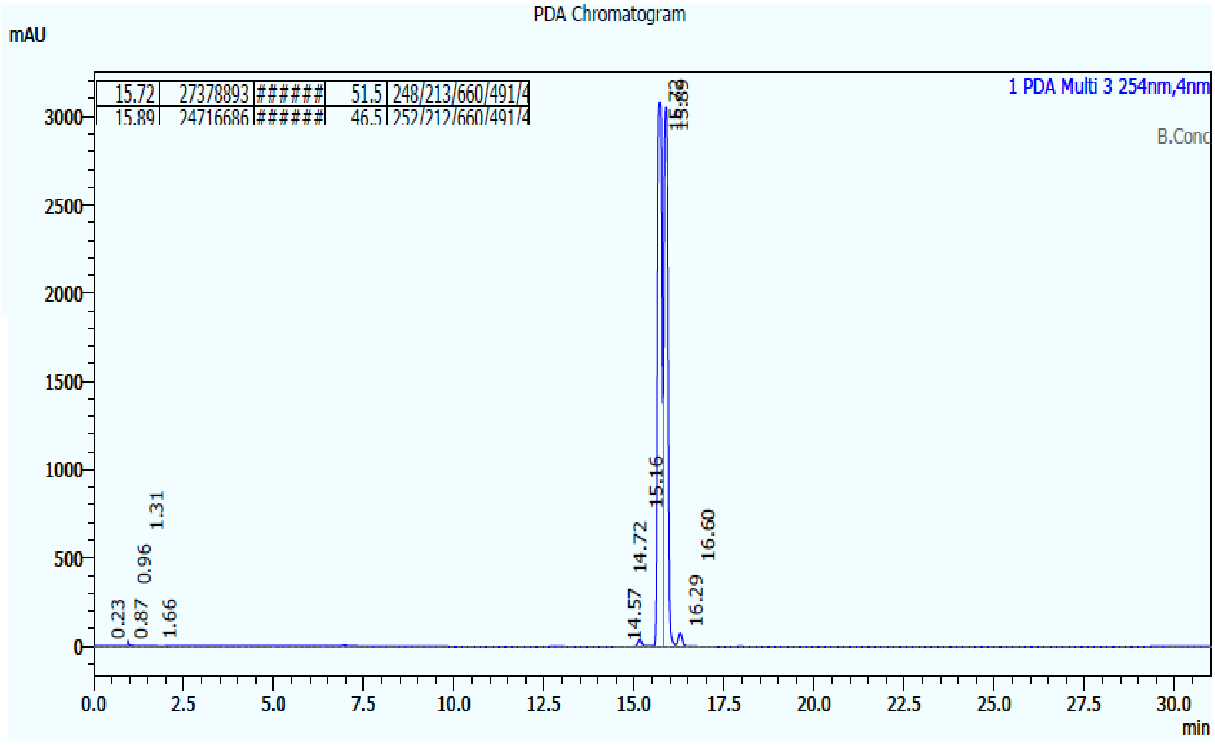

The HRMS spectrum of compound 16

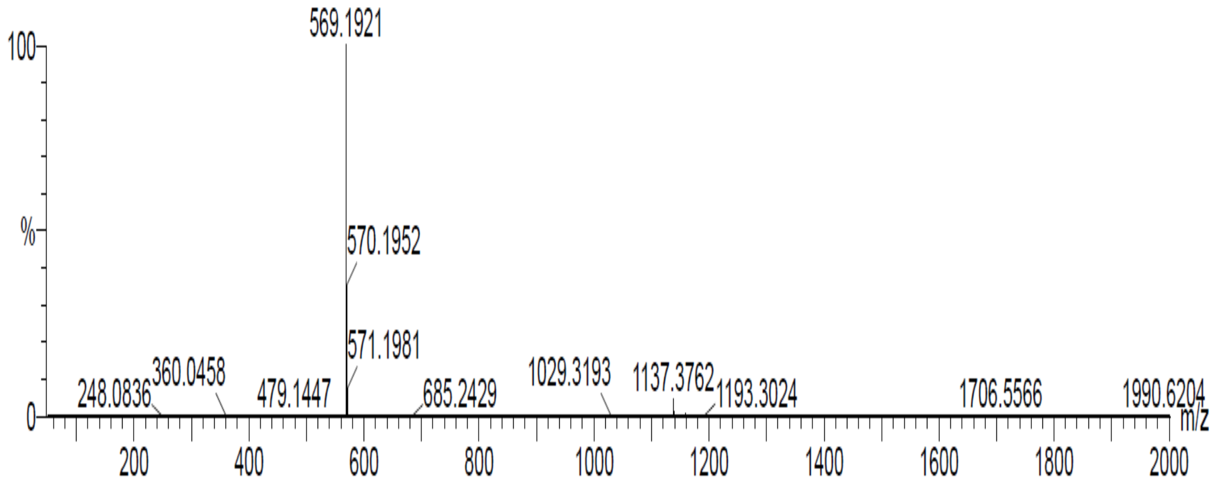

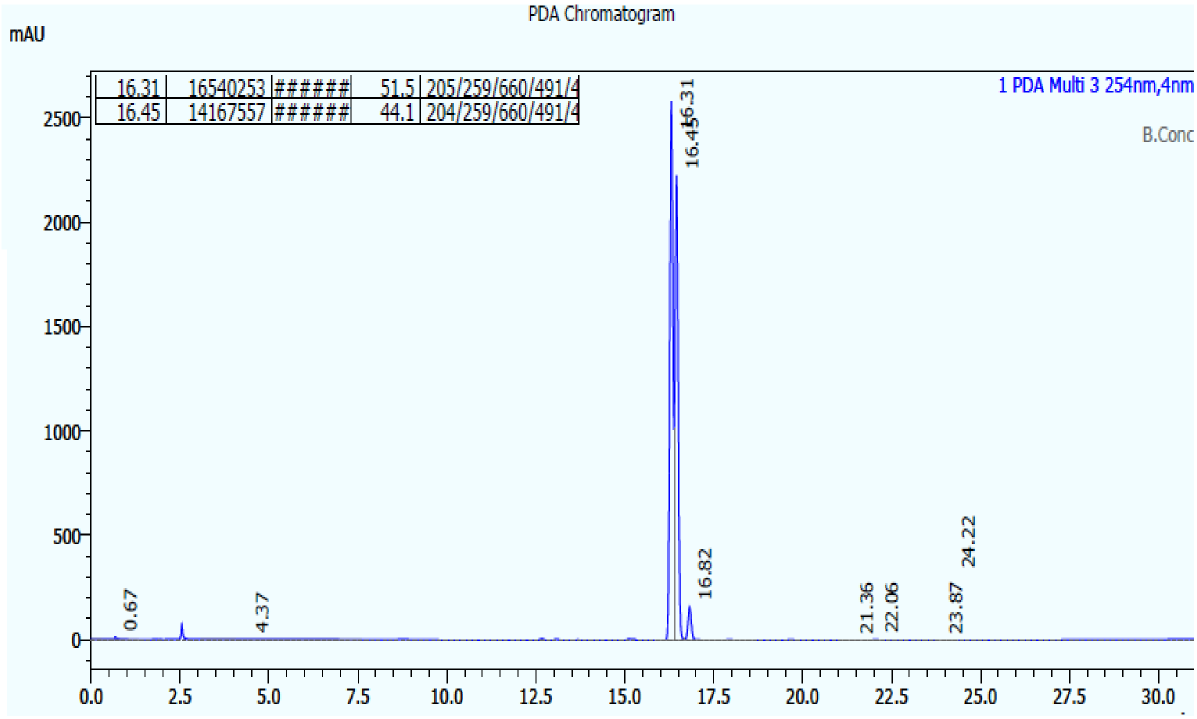

Supplement: Supplementary file 1 — Supplementary Material [file CMDC-21-e202501072-s001.pdf]
